# A fan effect in anaphor processing: effects of multiple distractors

**DOI:** 10.3389/fpsyg.2014.00818

**Published:** 2014-07-29

**Authors:** Kevin S. Autry, William H. Levine

**Affiliations:** Department of Psychological Science, University of ArkansasFayetteville, AR, USA

**Keywords:** comprehension, memory, fan effect, reading, anaphor resolution, antecedent, distractor

## Abstract

Research suggests that the presence of a non-referent from the same category as the referent interferes with anaphor resolution. In five experiments, the hypothesis that multiple non-referents would produce a cumulative interference effect (i.e., a fan effect) was examined. This hypothesis was supported in Experiments 1A and 1B, with subjects being less accurate and slower to recognize referents (1A) and non-referents (1B) as the number of potential referents increased from two to five. Surprisingly, the number of potential referents led to a decrease in anaphor reading times. The results of Experiments 2A and 2B replicated the probe-recognition results in a completely within-subjects design and ruled out the possibility that a speeded-reading strategy led to the fan-effect findings. The results of Experiment 3 provided evidence that subjects were resolving the anaphors. These results suggest that multiple non-referents do produce a cumulative interference effect; however, additional research is necessary to explore the effect on anaphor reading times.

## Introduction

Many theorists have argued that language comprehension processes can be explained in large part by appealing to general memory processes (e.g., Lewis, 1996; Gerrig and McKoon, 1998; Myers and O'Brien, 1998; Lewis and Vasishth, 2005; van den Broek et al., 2005); this hypothesis has been widely supported by empirical evidence. For example, general theories of memory processes have been shown to provide explanations for linguistic tasks such as establishing common ground between multiple parties (Horton and Gerrig, 2005) and resolving anaphors (O'Brien et al., 1990; Almor, 1999). Anaphor comprehension (often called anaphor resolution) in particular appears to rely heavily upon memory to determine co-reference between an anaphor and antecedent. Even within a sentence, limitations on working memory capacity induce the need for retrieval of referents (McElree, 2000). There are also instances, such as pronouns that refer to implicit referents (Greene et al., 1994) and bridging inferences (Garrod and Sanford, 1981), where anaphors are resolved even though the intended referent has not been explicitly mentioned. Such processes clearly rely on memory to produce an acceptable referent. Further evidence for the relationship between memory and anaphor resolution is provided by the findings that many factors affecting memory also affect anaphor resolution, including distance and elaboration (O'Brien et al., 1990), salience of the anaphor (Klin et al., 2004), salience of the referent (Foraker and McElree, 2007), and frequency (van Gompel and Majid, 2004). In the research reported here, we focus on anaphor resolution across sentences. Nevertheless, models of retrieval processes both across (Myers and O'Brien, 1998) and within (e.g., Lewis and Vasishth, 2005) sentences have many commonalities, which we highlight below.

Of particular interest for the current research are studies that have examined the effects of multiple potential referents on anaphor resolution (e.g., Corbett and Chang, 1983; Corbett, 1984; Mason, 1997; Levine et al., 2000; Wiley et al., 2001; Badecker and Straub, 2002; Klin et al., 2004, 2006; Ditman et al., 2007; Levine and Hagaman, 2008). In one of the first studies examining the effect of multiple potential referents, Corbett found longer reading time for an anaphoric noun phrase (e.g., *the frozen vegetable*) that included a category label when a text contained two members of that category (e.g., *fresh corn and frozen asparagus*) than when there was only a single category member (e.g., *frozen asparagus*). Badecker and Straub similarly found an increase in reading time shortly after subjects read reflexives when multiple gender-matched referents had been mentioned (e.g., *John thought that Bill owed himself another opportunity to solve the problem*). Levine et al. (see also Klin et al., 2004, 2006) found evidence suggesting that under some conditions anaphors (e.g., *the dessert*) appear not to be resolved at all when a text contains two potential referents from the same category (e.g., *tart* and *cake*), likely due to the increased difficulty in identifying a unique referent. The increased difficulty in processing anaphors in these studies suggests that readers engage in additional processing when a distractor (i.e., a non-referent) is present. Presumably this occurs because the both nouns are considered as potential referents, a process that is initiated by simple memory matching and that leads to retrieval-based interference. This explanation follows straightforwardly from global memory models (e.g., Ratcliff, 1978; Gillund and Shiffrin, 1984; Hintzman, 1986), which assume that stored memory representations that are related to a memory cue are activated in parallel and to the degree that they share features with the memory cue. Somewhat surprisingly, this additional processing appears to occur regardless of disambiguating material that should identify the proper referent, such as a prenominal adjective like *frozen* or the grammatical constraints that govern interpretation of reflexives (e.g., Reinhart, 1983). The reliability and time course of distractor interference, especially for within-sentence retrieval, is a matter of debate. Recent evidence is consistent with a very early role for grammatical constraints in retrieval. For example, Chow et al. (2014) were unable to replicate Badecker and Straub's results, and they found evidence that grammatical constraints prevent distractor interference (see also Dillon et al., 2013). Across sentence boundaries, some features, such as parallel structure (e.g., *Josh criticized Paul. Then Marie insulted him*.), may play an early role in limiting referent search (Chambers and Smyth, 1998). Nevertheless, for definite noun-phrase anaphors like *the dessert*, reported findings suggest that retrieval processes rely on semantic matching between an anaphor and potential referents, with no evidence as yet indicating that there are grammatical constraints on this process.

Whereas results like those from Badecker and Straub (2002), Corbett (1984), and Klin and colleagues (Levine et al., 2000; Klin et al., 2004, 2006) illustrate indirectly that distractors are considered during anaphor resolution, direct evidence that distractors are activated during anaphor resolution comes from results reported by O'Brien et al. (1990). O'Brien et al. had subjects read passages with two potential antecedents (e.g., *train* and *plane*), which appeared early and late in a passage and were sometimes described elaborately. At the end of a passage, a sentence (e.g., *Mark's neighbor asked him how he had traveled to his parent's*) appeared that required retrieval of only one of the antecedents. Following this sentence, subjects had to name aloud one of the potential antecedent nouns. Relative to a no-anaphor control condition, referent nouns were named more quickly, replicating findings that suggest that referents are activated by anaphor resolution processes (e.g., Dell et al., 1983). Of perhaps greater interest was the finding that non-referent concepts were also activated relative to a control condition, especially when they were elaborated and appeared in the late position in the passage, between the anaphor and the correct antecedent. These results are consistent with the hypothesis that an anaphor acts like any other memory cue, activating related information in parallel. The finding that non-referent concepts were activated, especially when they occurred late and were elaborated, again fits very well with well-established findings from the memory literature that recency and elaboration lead to easier memory access.

Taken together, these studies demonstrate that people consider multiple potential referents when resolving anaphors, and further, that the resolution of the anaphor increases activation for the referent. However, studies involving distractors have typically been limited to situations with a single distractor. Therefore, the effect of additional distractors remains an open empirical question. A yet-stronger case that general memory processes govern anaphor resolution can be made if there is a cumulative effect of additional distractors. Both Myers and O'Brien's (1998) resonance model and Lewis and Vasishth's (2005) implementation of ACT-R (e.g., Anderson, 2005) as a theory of memory-retrieval in sentence-processing make similar predictions about the effect of multiple distractors. The resonance model states that elements in the mental representation resonate to signals from retrieval cues. In the case of anaphor resolution, the retrieval cue is the anaphor and the resonating elements are related items in the mental representation. Critically, the signal (i.e., resonance strength) of any item in the representation is divided among receiving elements, and only a subset of the elements with the strongest signal enter working memory (WM). Thus, the strength of a referent will be reduced in the presence of related distractors, reducing the probability that the correct referent will be selected into WM. Similarly, Lewis and Vasishth's model states that the activation that a chunk in memory will receive is reduced as there are more chunks in memory associated with a particular cue. Given the assumption that activation determines retrieval latency and the probability of the retrieval of a memory chunk, there should be greater difficulty in retrieving the correct referent with every additional distractor.

We can also draw on the memory literature to provide empirical guidance about the possible effects of multiple distractors. Specifically, research has shown that reading sentences that pair a person with multiple locations (or a location with multiple people) slows later recognition of the sentences (Anderson, 1974; Radvansky, 1998; Anderson and Reder, 1999). This result, known as the fan effect, is hypothesized to occur because of interference among competing associations in memory. Unlike the anaphor literature, which has focused on single distractors, the fan effect literature has explored situations with more than two associations and has demonstrated a cumulative effect, such that additional associations cause additional interference.

In the original demonstration of the fan effect (Anderson, 1974), subjects studied sentences in which a person was paired with a location (see 1–4 below).

1. A hippie is in the park.
2. A hippie is in the church.
3. A policeman is in the park.
4. A sailor is in the park.

Importantly, some people were associated with more than one location and some locations were associated with more than one person. For example, the sailor was associated only with the park (i.e., a fan of one), the hippie was associated with both the park and the church (i.e., a fan of two), and the park was associated with hippie, the policeman, and the sailor (i.e., a fan of three). Thus, the nouns varied in the number of associations with other nouns. After the study phase, subjects read another set of sentences, some of which were the same as those studied previously and some of which were novel pairings of people and locations that the subjects had not seen. For each sentence, subjects indicated whether it was the same as one they had read during the study phase or not. Consistent with the hypothesis that multiple associations interfere with one another, subjects were slower to recognize sentences with nouns that were associated with more nouns compared to sentences with nouns associated with fewer nouns. That is, subjects were slower to respond as the size of the noun's fan increased.

If anaphor resolution relies on general memory processes, and increasing the number of associations with a noun increases interference, then we can predict that increasing the total number of potential referents for an anaphor should also show a cumulative retrieval-interference effect (i.e., a fan effect). The present study tested this prediction across five experiments by exploring the effects of multiple distractors on anaphor resolution and the subsequent activation levels of referents and distractors. In particular, we used a probe recognition task after anaphor sentences to measure the relative activation of an anaphoric referent when there were a variable number of distractors. We also used the probe task to measure activation of those distractors as a function of the number of distractors. Our results demonstrate evidence of a fan effect in anaphor resolution.

## Experiment 1A

In Experiment 1A, subjects read pairs of sentences. The first provided an antecedent and one or more distractors in a serial list, and the second included an anaphoric noun phrase that co-referred with the antecedent; these were followed by a probe recognition task that was used to measure the activation of the referent concept (see Table 1 for a sample passage and Appendix A in Supplementary Materials for a full list of experimental passages). In particular, the first sentence ended with a list of two, three, four, or five potential referents from the same taxonomic category, and the second sentence referred with a disambiguating adjective and categorical anaphor to a single item mentioned in the list. Following each sentence-pair, subjects completed a probe recognition task to measure the activation level of the referent following the anaphor. For example, the first sentence in the example in Table 1 describes a person looking through a toolbox with a number of tools in it. The last tool mentioned in the sentence, a saw, is the antecedent concept. The second sentence then describes the person fixing a table using *the cutting tool*. The latter noun phrase serves as an unambiguous reference to the entity introduced by the antecedent. After the second sentence was completed, the word *saw* was presented in an old-new recognition task, the correct response for which is “old.” We assume that reaction time and accuracy in responding to the probes will reflect the ease or difficulty the subjects have in selecting the correct referent (cf. Dell et al., 1983; Levine et al., 2000) from the list of potential referents, including the distractors and the referent.

**Table 1:** **Sample passage**.

List sentence	Amelia's new table was wobbling, so she looked in her toolbox and found …
Two-noun	… a hammer and a saw. (all experiments)
Three-noun	… a screwdriver, a hammer, and a saw. (Experiments 1A and 1B only)
Four-noun	… a level, a screwdriver, a hammer, and a saw. (Experiments 1A and 1B only)
Five-noun	… a wrench, a level, a screwdriver, a hammer, and a saw. (all experiments)
**REFERENCE SENTENCE**	
Anaphor	She fixed it with the cutting tool before it broke. (all experiments)
No anaphor	She fixed the table all by herself before it broke. (Experiment 3 only)
**PROBE WORD**	
Referent	SAW (Experiments 1A, 2A, 2B, and 3)
Distractor	HAMMER (Experiments 1B, 2A, and 2B)
Comprehension question	Did Amelia use the saw? (all experiments)

We hypothesized that increasing the number of distractors would lead activation from the anaphor to spread among the referent and distractor concepts (Kintsch, 1988; Myers and O'Brien, 1998; Lewis and Vasishth, 2005). It was expected that the spread of activation from the anaphor to all conceptually-related potential referents would cause the referent to be less active following anaphor resolution as the number of distractors increased (i.e., a monotonic increasing trend in reaction time and decreasing trend in accuracy was expected), resulting in lower probe accuracy and longer probe recognition times. Additionally, this spread of activation should interfere with the selection of the appropriate referent during anaphor resolution, thus slowing reading of the reference sentence, replicating several findings (e.g., Corbett and Chang, 1983; Corbett, 1984; Mason, 1997; Badecker and Straub, 2002). Alternatively, it is possible that a backward, parallel-search process occurs such that the earlier-occurring distractors have little or no detectable impact on anaphor resolution (O'Brien, 1987). A backward, serial, self-terminating search would also predict no impact of early distractors on resolution of later referents. This latter strategy seems attractive especially in short passages with a list-like first sentence (cf. Townsend and Fifíc, 2004).

### Method

#### Subjects

Ninety-five students enrolled in a general psychology course at the University of Arkansas participated in the experiment to partially fulfill a research requirement. All subjects were native-English speakers. Informed consent was obtained from all subjects in this and all subsequent experiments.

#### Materials and design

There were 31^1^ experimental sentence-pairs that appeared in one of four conditions (see Table 1). Each sentence-pair began with a list sentence that introduced a character by proper name (half stereotypically male, half stereotypically female) and ended in a list of either two, three, four, or five nouns from the same taxonomic category. The nouns were all single words, common, and were selected to be roughly equal in typicality as judged by the first author and several research assistants. Furthermore, each of the last two nouns in the list was able to be distinguished from the other nouns by means of an adjective (e.g., saws can be distinguished from the other tools in the list using the adjective *cutting*). The list sentence was followed by a reference sentence that unambiguously referred to the final item in the list using an adjective and a categorical anaphor (e.g., *cutting tool*) that was the same for all conditions. The anaphor always occurred three words prior to the end of the reference sentence to ensure that there was enough time for the anaphor to be resolved by the time the sentence was fully read (i.e., by the time the probe-word task was presented).

In addition, there were 68 filler sentence-pairs that each included a list sentence with two to five nouns but that were not limited by the same restrictions on nouns in the experimental lists (e.g., the nouns could be proper or multiple words). As with the experimental sentence pairs, the filler reference sentences also included a categorical anaphor modified by an adjective; however, the referent of the anaphor was not always completely unambiguous. Moreover, the referent of the anaphor was not always the last item in the list. These two features of the fillers were expected to encourage subjects to put forth more effort in resolving anaphors across all trials.

Each experimental and filler sentence-pair also had a corresponding recognition probe and comprehension question. Following the reference sentence, subjects completed a probe recognition task in which they indicated whether a word on the screen had occurred in the previous sentence-pair. For experimental sentence-pairs, the probe word was always the final noun from the list, which required a “yes” response. To ensure an equal number of “yes” and “no” responses across the experiment, the majority of the filler probe tasks presented a word that did not occur in the sentence pair and therefore required a “no” response. Other fillers presented a probe word that was not the final noun from the list, requiring a “yes.” Finally, a comprehension question was presented following the probe recognition task, half of which required a “yes” response and half of which required a “no” response. Comprehension questions frequently, but not always, focused on correct resolution of the anaphor (e.g., *Did Amelia use the saw?*).

Subjects saw each experimental sentence-pair in one of the four conditions along with all filler sentence-pairs. Four counterbalanced lists were created with the following constraints: one quarter of the list sentences had two nouns, one quarter had three nouns, one quarter had four nouns, and one quarter had five nouns. Furthermore, a second set of materials^2^ was created that reversed the order of the final two nouns in the list, such that final noun in the first set of materials (e.g., *saw*) became the penultimate noun and the formerly penultimate noun (e.g., *hammer*) became the final noun. This also required a change in the disambiguating adjective in the reference sentence (e.g., *cutting* changed to *pounding*) such that the referent of the categorical anaphor was always the final noun. The manipulation of these factors resulted in a design that was 4 (nouns: 2, 3, 4, 5) × 2 (noun order: order 1, order 2).

#### Procedure

The experiment began with three practice blocks of five trials each, which were intended to familiarize the subject with the yes/no response keys, the probe recognition task, and the comprehension question, respectively. For all practice trials, feedback about the correctness of subjects' responses was provided.

Subjects then began the experimental session. Subjects were instructed to read the sentences as they normally would for comprehension and to respond to the probe words as quickly and accurately as possible. Each trial consisted of a list sentence, a reference sentence, a probe word, and a comprehension question. At the beginning of each trial, subjects were given the instruction “PRESS THE SPACEBAR WHEN READY” centered on a computer monitor. When they pressed the spacebar, the list sentence appeared left-justified in the middle of the screen. Subjects pressed the spacebar to indicate when they had finished reading the list sentence, which removed the list sentence from the screen and replaced it with the reference sentence. Subjects pressed the spacebar again to indicate when they had finished reading the reference sentence, which removed the reference sentence from the screen and replaced it with a probe word in all capital letters in the center of the screen. Subjects used the left and right arrow keys labeled “Y” and “N” for yes and no, respectively, to respond to the probe task. This removed the probe word and replaced it with a comprehension question in the center of the screen; no feedback about correctness was provided for probes or questions. Subjects again used the yes and no keys to respond to the comprehension question, which ended the trial.

The experimental session consisted of 99 trials (31 experimental and 68 fillers) in three blocks of 25 trials and one block of 24 trials. The order of the blocks, as well as the order of the trials within each block, was randomized with the restriction that the first sentence-pair of each block was always a filler sentence-pair, to allow time for the subjects to fully return their attention to the task after a mandatory 10 s break between blocks. Subjects were free to take breaks between trials. The experiment lasted approximately 30 min. The procedure for this and all subsequent experiments were approved by the University of Arkansas Institutional Review Board.

### Results

#### Data exclusion and general analytic considerations

A subject's data were excluded from further analysis if they met any of the following criteria: (1) they had more than 30% of reading times less than 1000 ms or greater than 7500 ms; (2) they had lower than 70% probe recognition accuracy; (3) they had more than 30% of probe reaction times less than 500 ms or greater than 2500 ms; (4) they had no non-outlying probe recognition observations in at least one condition; or (5) they had less than 70% comprehension question accuracy. Based on these criteria, the data from eight subjects were excluded from further analysis. Additionally, two experimental items were removed from further analysis due to counterbalancing errors. Therefore, the reported analyses include 85 subjects and 29 items.

For all experiments reported in this paper, subject and item condition means were analyzed separately; a subscript of 1 indicates that subjects were treated as a random-effects variable, whereas a subscript of 2 indicates that items were treated as a random-effects variable. For all significance tests, an alpha level of 0.05 was used. Predictions about monotonic increasing and decreasing trends were tested using polynomial contrasts. For all repeated-measures effects with more than one numerator *df*, Huynh-Feldt adjusted *p*-values are reported to correct for sphericity violations. Effect-size measures that are reported are based on the subject analyses, and all within-subject standard errors in figures and tables were computed using the method recommended by Loftus and Masson (1994).

#### Comprehension

In general, the number of nouns did not affect comprehension (see Table 2 for comprehension results across all experiments). The linear trend was non-significant, *F*_1(1, 84)_ = 0.34, *p* = 0.56, *F*_2(1, 56)_ = 0.07, *p* = 0.79, with no significant higher-order trends. (See Appendix C in Supplementary Materials for the results of the noun-order factor in this experiment and Experiment 1B.)

**Table 2:** **Mean comprehension for all experiments (with standard errors of the mean)**.

	**Two-noun**	**Three-noun**	**Four-noun**	**Five-noun**
Experiment 1A	0.88 (0.014)	0.86 (0.014)	0.87 (0.014)	0.87 (0.014)
Experiment 1B	0.89 (0.014)	0.87 (0.015)	0.83 (0.017)	0.86 (0.016)
Experiment 2A				
Referent	0.95 (0.012)	–	–	0.92 (0.015)
Distractor	0.93 (0.012)	–	–	0.91 (0.014)
Experiment 2B				
Referent	0.93 (0.013)	–	–	0.88 (0.021)
Distractor	0.92 (0.015)	–	–	0.91 (0.015)
Experiment 3				
Anaphor	0.88 (0.019)	–	–	0.90 (0.014)
No anaphor	0.96 (0.011)	–	–	0.91 (0.015)

#### Probe accuracy

**Figure 1:**
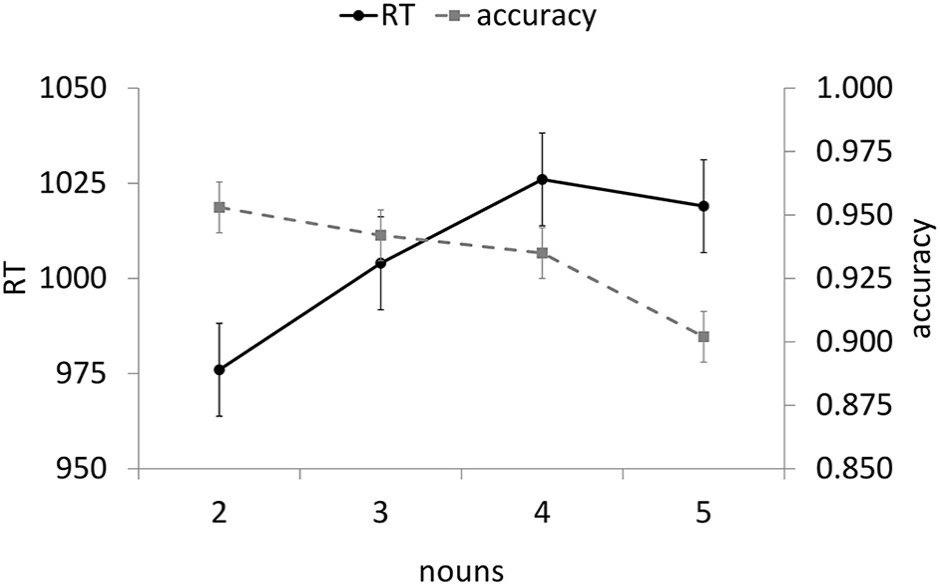
**Experiment 1A antecedent probe reaction times and accuracies by noun condition (error bars indicate SE of the mean)**.

Figure 1 presents mean probe word accuracy and reaction times along with mean reference-sentence reading times as a function of the number of referents. In general, accuracy decreased as the number of nouns in the list sentence increased. The linear trend was significant, *F*_1(1,84)_ = 9.63, *p* = 0.003, *F*_2(1,28)_ = 9.99, *p* = 0.004, η^2^_*p*_ = 0.10, with no significant higher-order trends.

#### Probe reaction times (RT)

Only correct probes were analyzed. Outliers were first classified as RTs that were less than 400 ms or greater than 3000 ms. Remaining reaction times more extreme than 1.5 times the interquartile range above the 75th percentile or below the 25th percentile for each subject were classified as outliers (Tukey, 1977), resulting in 8.6% of the data being excluded from further analyses. In general, reaction time increased as the number of nouns in the list sentence increased (see Figure 1). The linear trend was significant, *F*_1(1, 84)_ = 8.03, *p* = 0.006, *F*_2(1,28)_ = 6.68, *p* = 0.02, η^2^_*p*_ = 0.09, with no significant higher-order trends.

#### Reference-sentence reading times

Reference-sentence reading times were transformed to per-character reading times by dividing the full-sentence reading time by the number of characters in the sentence, not counting spaces and punctuation (see Table 3). Outliers were first identified as trials with less than 15 ms/char or more than 150 ms/char. Outliers among the remaining reading times were then identified within each subject based on Tukey's (1977) criteria. 7.6% of the trials were excluded from further analysis. In general, reading time on the reference sentence *decreased* as the number of nouns in the list sentence increased. The linear trend was significant, *F*_1(1,84)_ = 19.55, *p* < 0.001, *F*_2(1,30)_ = 10.87, *p* = 0.003, η^2^_*p*_ = 0.19, with no significant higher-order trends.

**Table 3:** **Experiment 1A mean per-character reading times in ms (with standard errors of the mean)**.

	**List sentence**	**Reference sentence**
Two-noun	73.2 (0.9)	58.3 (0.7)
Three-noun	74.0 (0.9)	57.5 (0.7)
Four-noun	76.8 (0.9)	55.4 (0.7)
Five-noun	77.4 (0.9)	53.9 (0.7)

### Discussion

The results of the probe word analyses were consistent with the fan-effect hypothesis and generally favor models of anaphor resolution that posit a parallel-search mechanism in retrieval of the correct referent. As predicted, the presence of distractors interfered with the probe recognition task. Increasing the number of distractors in the list sentence decreased recognition accuracy and increased reaction times for referents, which suggests that the activation level of referents decreased as the number of distractors increased. The existing literature has shown via a variety of measures and paradigms that the presence of one distractor interferes with anaphor resolution (e.g., Corbett and Chang, 1983; Corbett, 1984; Mason, 1997; Levine et al., 2000; Wiley et al., 2001; Klin et al., 2004, 2006; Ditman et al., 2007; Levine and Hagaman, 2008). The present results extend this finding by demonstrating a cumulative effect of distractors.

The effect of additional nouns on the subsequent reference-sentence reading times, however, was unexpected. It was predicted, based on previous research (e.g., Corbett, 1984), that anaphor resolution would be slowed by the presence of distractors, resulting in longer full-sentence reading times as the number of distractors increased. However, the results were exactly the opposite, indicating that the subjects actually read the reference sentences more quickly as the number of distractors increased. Assuming this is not a Type I error, one plausible explanation for this result is that subjects adopted a strategy of speeding through the reference sentence to reduce the time between the referents and the probe recognition task when there were more distractors. A similar finding was reported by Van Dyke and McElree (2006), who had subjects reading sentences of variable complexity while holding or not holding a memory load and found that reading was faster for more-complex sentences with a memory load than without one. This speeded-reading strategy as a potential alternative explanation for the fan effect was explored in further detail in Experiments 2A and 2B; we defer discussion until the presentation of those experiments.

## Experiment 1B

Experiment 1A established that referents were less active following anaphor resolution when there were more potential referents available in the discourse. Experiment 1B replicated Experiment 1A but used distractors as the probe words to test the effect of multiple distractors on the activation level of a distractor. As in Experiment 1A, it was hypothesized that additional distractors would decrease probe accuracy and slow probe recognition times. If anaphors act like any other cue to memory, the activation of the referent and distractors should be split (Kintsch, 1988; Myers and O'Brien, 1998; Lewis and Vasishth, 2005), resulting in less activation to go around (i.e., a fan effect) as there are more related concepts in the list sentence. Because the anaphor contains two cues (i.e., adjective plus noun) to retrieve the referent but only one (i.e., the noun) that matches the distractors, referents should become more active and experience less interference (i.e., a reduced fan effect) than distractors following anaphor resolution. Moreover, later items may overwrite or displace earlier items, leading to degraded representations of the referent and especially earlier-occurring distractors (Nairne, 1990; Lewis, 1996). We examine these predictions in a cross-experiment comparison after presenting the results of Experiment 1B, and then examine them more directly (i.e., in a completely within-subjects design) in Experiments 2A and 2B.

### Method

#### Subjects

Seventy-eight students enrolled in a general psychology course at the University of Arkansas participated in the experiment to partially fulfill a research requirement. All subjects were native-English speakers.

#### Materials, design, and procedure

Experiment 1B was identical to Experiment 1A except that the probe words in the probe recognition task for experimental trials were distractors (i.e., the penultimate word in the list).

### Results

#### Data exclusion and general analytic considerations

Based on the data exclusion criteria detailed in Experiment 1A, the data from eight subjects were excluded from further analysis. Sixteen more subjects were removed from further analysis for a systematic misunderstanding of the instructions. These subjects consistently responded “no” to distractors on the probe task when they should have been responding “yes.” This pattern of responding suggests that these subjects were correctly identifying the correct referent of the anaphor, but misunderstanding that this identification was unrelated to the probe task. Therefore, the comprehension accuracy, probe accuracy, and reading time analyses included 54 subjects and 31 items.

#### Comprehension

In general, comprehension (see Table 2) decreased as the number of nouns increased. The linear trend was significant in the subject analysis, *F*_1(1, 53)_ = 5.36, *p* = 0.025, η^2^_*p*_ = 0.09, but non-significant in the items analysis, *F*_2(1, 60)_ = 2.91, *p* = 0.093, with no significant higher-order trends.

#### Probe accuracy

**Figure 2:**
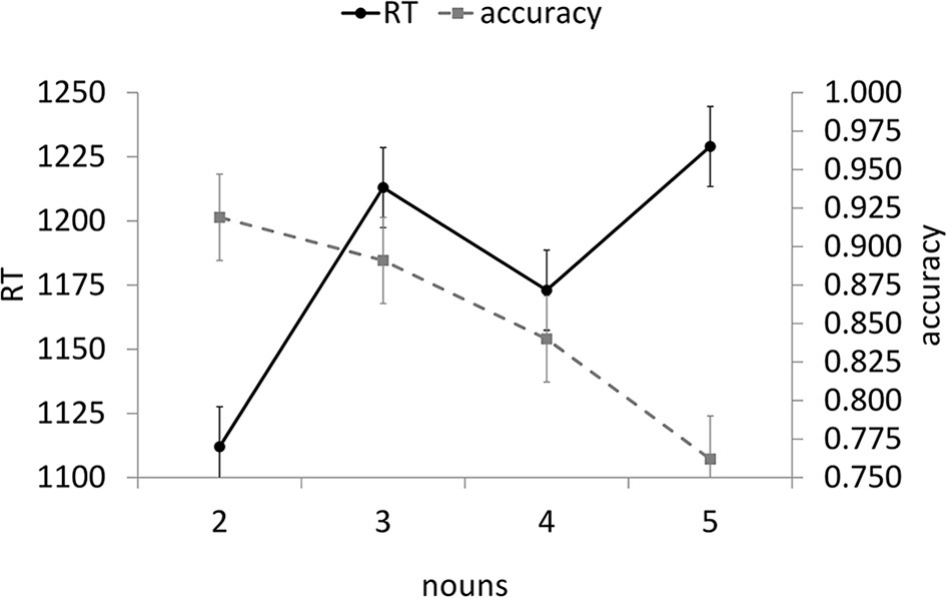
**Experiment 1B distractor probe reactions times and accuracies by noun condition (error bars indicate SE of the mean)**.

Figure 2 presents mean probe word accuracy and reaction times along with mean reference-sentence reading times as a function of the number of referents. In general, accuracy decreased as the number of nouns in the list sentence increased. The linear trend was significant, *F*_1(1, 53)_ = 39.08, *p* < 0.001, *F*_2(1, 30)_ = 45.28, *p* < 0.001, η^2^_*p*_ = 0.42, with no significant higher-order trends.

#### Probe reaction times

Based on outlier exclusion criteria, 9.6% of the data were excluded from further analyses. In general, reaction time increased as the number of nouns in the list sentence increased (see Figure 2). The linear trend was significant, *F*_1(1, 53)_ = 16.79, *p* < 0.001, *F*_2(1, 30)_ = 6.59, *p* = 0.01, η^2^_*p*_ = 0.24. There was also an unexpected cubic trend, *F*_1(1, 53)_ = 12.81, *p* = 0.001, *F*_2(1, 30)_ = 3.13, *p* = 0.09. There was no theoretical expectation of this effect, and it did not appear in Experiment 1A, so we did not try to interpret it.

#### Reference-sentence reading times

Based on outlier exclusion criteria, 5.2% of the data were excluded from further analyses. In general, as in Experiment 1A, reading time (see Table 4) on the reference sentence decreased as the number of nouns in the list sentence increased. The linear trend was significant, *F*_1(1,53)_ = 11.74, *p* = 0.001, *F*_2(1, 30)_ = 11.52, *p* = 0.002, η^2^_*p*_ = 0.18, with no significant higher-order trends.

**Table 4:** **Experiment 1B mean per-character reading times in ms (with standard errors of the mean)**.

	**List sentence**	**Reference sentence**
Two-noun	77.0 (1.11)	60.9 (0.88)
Three-noun	80.3 (1.11)	59.8 (0.88)
Four-noun	80.3 (1.11)	58.3 (0.88)
Five-noun	82.5 (1.11)	57.4 (0.88)

### Discussion

The probe word results were again consistent with the fan-effect hypothesis. As predicted, the presence of distractors interfered with the probe recognition task. Increasing the number of referents in the list sentence decreased recognition accuracy and increased reaction times for distractors similar to the effect found for referents in Experiment 1A. This result extends the findings of Experiment 1A to show that distractors also decrease in activation as the number of referents increases.

As in Experiment 1A, the reading-time results did not support the fan-effect hypotheses. Subjects again read the reference sentence more quickly as the number of distractors increased. This replication provides additional confidence that the unexpected results were not occurring due to chance. This issue was explored in further detail in Experiments 2A and 2B.

## Experiments 1A and 1B combined analysis

As noted in the introduction to Experiment 1B, the effect of fan size should be different for referents (Experiment 1A) and distractors (Experiment 1B). To compare the magnitude of the effect of the number of nouns on referents and distractors, an additional analysis was conducted for the probe reaction times from Experiments 1A and 1B. Probe reaction times for each subject in both experiments were first linearly regressed on the number of nouns (cf. Lorch and Myers, 1990), and the slopes were then examined in an independent-samples *t*-test with experiment (i.e., probe: referent vs. distractor) as a between-subjects variable. This analysis revealed a non-significant effect of probe in the expected direction, with a substantially smaller mean slope among subjects responding to referents in Experiment 1A (*M*_slope_ = 15.2 ms/noun, *SE* = 5.4) than among subjects responding to distractors in Experiment 1B (*M*_slope_ = 31.3 ms/noun, *SE* = 7.6), *t*_(137)_ = 1.77, *p* = 0.08, *d* = 0.30.

A similar analysis performed on the accuracy data revealed a large and significant effect of probe, with a substantially smaller mean slope among subjects responding to referents in Experiment 1A (*M*_slope_ = −0.014 accuracy/noun, *SE* = 0.0046) than among subjects responding to distractors in Experiment 1B (*M*_slope_ = −0.052 accuracy/noun, *SE* = 0.0084), *t*_(137)_ = 4.33, *p* < 0.001, *d* = 0.74. Although referents likely gained an advantage in both accuracy and speed of responding due to having appeared more recently than distractors, these analyses focused on the linear trends in which distance from the probe were equal. Therefore, these results provide evidence that the interference effect is greater for distractors than referents; this effect was tested more directly in Experiments 2A and 2B.

## Experiment 2A

The procedure for Experiment 2A was modified from that in Experiments 1A and 1B such that subjects read the reference sentence one word at a time. This allowed for a more detailed analysis of the reading times, which was necessary to help understand the unexpected reference-sentence reading time results of Experiments 1A and 1B. The prediction that additional distractors should slow reading of the reference sentence was based on the hypothesis that multiple distractors would interfere with anaphor resolution. This means that the expected slowdown should occur specifically on the anaphor or immediately after the anaphor during spillover processing. According to this hypothesis, it was expected that there should be no difference in reading times on the reference-sentence until subjects reach the anaphor and post-anaphor regions, where they were expected to read more slowly as the number of distractors increased. However, if the results of Experiments 1A and 1B are reliable, then there should be longer reading times when there are more distractors at some point in the reference sentence prior to the anaphor.

In addition, Experiments 1A and 1B demonstrated that the presence of multiple distractors made recognition of both referents and distractors more difficult, as indexed by both reaction time and accuracy. Experiments 2A and 2B were designed to manipulate the probe word within subjects to address potential concerns about comparing results across experiments. Thus, in these experiments, probe word (referent vs. distractor) and number of distractors (two vs. five) were manipulated within subjects. The fan-effect hypothesis predicts that additional distractors would slow recognition and decrease accuracy for both referents and distractors. Moreover, to the extent that anaphor resolution focuses activation on the referent, thereby minimizing interference, the degree of interference should be greater for distractors than for referents.

### Method

#### Subjects

Seventy-five students enrolled in a general psychology course at the University of Arkansas participated in the experiment to partially fulfill a research requirement. All subjects were native-English speakers.

#### Materials and design

Thirty of the experimental materials from Experiment 1 were used and appeared in only the two- and five-noun list conditions. This also required some modification of the list length in the filler sentences to maintain an equal distribution of list lengths across the entire experiment. In addition, the probe words were manipulated within subjects, such that each subject saw an equal number of referent and distractor probes following experimental items.

Subjects saw each experimental sentence pair in one of the four conditions along with all filler sentence pairs. Four counterbalanced lists were created with the following constraints: approximately (i.e., 7 or 8 items) one quarter of the list sentences had two nouns followed by a referent probe, approximately one quarter had two nouns followed by a distractor probe, approximately one quarter had five nouns followed by a referent probe, and approximately one quarter had five nouns followed by a distractor probe. Because counterbalancing order did not have any important effects in Experiments 1A and 1B, order was no longer manipulated, resulting in a 2 (nouns: 2, 5) × 2 (probe word: referent, distractor) completely within-subjects design.

#### Procedure

The experiment was conducted using Linger (Rohde, 2003) to present the materials using a moving window (Just et al., 1982). Before starting the experiment, subjects completed three practice trials to familiarize themselves with the procedure. Each trial began with two rows of dashes, centered on the left-hand side of the screen, with each dash replacing a character or space in the sentences. Subjects pressed the spacebar to initially present the list sentence in its entirety. When they finished reading the list sentence, subjects pressed the spacebar again which replaced the list sentence with dashes and revealed the first word of the reference sentence. Subjects continued to press the spacebar to advance from one word to the next, with each press replacing the previous word with dashes and revealing the next word in the sentence. Pressing the spacebar after the final word of the reference sentence removed all of the dashes from the screen and presented a probe word in all capital letters in the center of the screen. Subjects responded to the probe word using the F key for yes and the J key for no. The response removed the probe word from the screen and replaced it with a comprehension question. Subjects again responded using the F and J keys, which advanced the screen to the next trial.

The experimental session consisted of 98 trials (30 experimental and 68 fillers) in two blocks of 49 trials each with the order of the trials completely randomized. Subjects were instructed to read the sentences as they normally would for comprehension and to respond to the probe words as quickly and accurately as possible. Subjects were free to take breaks between trials. The experiment lasted approximately 30 min.

### Results

#### Data exclusion and general analytic considerations

Based on the data exclusion criteria, the data from six subjects were excluded from further analysis. Therefore, the reported analyses included 69 subjects and 30 items.

#### Comprehension

In general, comprehension (see Table 2) decreased as the number of nouns increased. A 2 (nouns: 2, 5) × 2 (noun probed: referent, distractor) repeated-measures ANOVA revealed a main effect of nouns that was non-significant in the subject analysis, *F*_1(1, 68)_ = 3.38, *p* = 0.07, η^2^_*p*_ = 0.05, but significant in the items analysis, *F*_2(1, 29)_ = 4.82, *p* = 0.04. The main effect of noun probed was non-significant, *F*_1(1, 68)_ = 2.62, *p* = 0.11, *F*_2(1, 29)_ = 1.07, *p* = 0.31, and the interaction between number of nouns and noun probed was also non-significant, *F*_1(1, 68)_ = 0.01, *p* = 0.92, *F*_2(1, 29)_ = 0.14, *p* = 0.71.

#### Probe accuracy

Table 5 presents mean accuracy and probe reaction times as a function of the number of nouns and the noun probed. In general, accuracy was higher for referents than for distractors and when there were two nouns in the list sentence than when there were five. A 2 (nouns: 2, 5) × 2 (noun probed: referent, distractor) repeated-measures ANOVA revealed a significant main effect of the number of nouns, *F*_1(1, 68)_ = 28.34, *p* < 0.001, *F*_2(1, 29)_ = 25.46, *p* < 0.001, η^2^_*p*_ = 0.29, as well as a significant main effect of the noun probed, *F*_1(1, 68)_ = 17.62, *p* < 0.001, *F*_2(1, 29)_ = 28.98, *p* < 0.001, η^2^_*p*_ = 0.21. There was also a significant interaction between the number of nouns in the sentence and the noun being probed, *F*_1(1, 68)_ = 4.51, *p* = 0.04, *F*_2(1, 29)_ = 4.37, *p* = 0.05, η^2^_*p*_ = 0.06, with a greater 2- vs. 5-noun difference for distractors than for referents, replicating the effect seen in the between-experiments comparison presented above. Planned pairwise comparisons revealed a significant effect of the number of nouns for both the referent probes, *t*_1(68)_ = 3.04, *p* = 0.003, *t*_2(29)_ = 3.69, *p* = 0.001, *d* = 0.37, and the distractor probes, *t*_1(68)_ = 4.53, *p* < 0.001, *t*_2(29)_ = 4.17, *p* < 0.001, *d* = 0.55.

**Table 5:** **Experiments 2A and 2B mean probe word responses (with standard errors of the mean)**.

	**Experiment 2A**			
	**Accuracy**		**Reaction time (ms)**	
	**Referent**	**Distractor**	**Referent**	**Distractor**
Two-noun	0.97 (0.013)	0.92 (0.013)	1553 (25.1)	1632 (25.1)
Five-noun	0.93 (0.013)	0.83 (0.013)	1599 (25.1)	1705 (25.1)
	**Experiment 2B**			
	**Accuracy**		**Reaction time (ms)**	
	**Referent**	**Distractor**	**Referent**	**Distractor**
Two-noun	0.95 (0.017)	0.92 (0.019)	1151 (21.5)	1273 (21.5)
Five-noun	0.94 (0.013)	0.72 (0.022)	1245 (21.5)	1403 (21.5)

#### Probe reaction times

Based on outlier exclusion criteria, 7.8% of the data were excluded from further analyses. Like the accuracy results, reaction time tended to be shorter for referents than for distractors and when there were two nouns in the list sentence than when there were five. A 2 (nouns: 2, 5) × 2 (noun probed: referent, distractor) repeated-measures ANOVA revealed a significant main effect of the number of nouns, *F*_1(1, 68)_ = 4.20, *p* = 0.04, *F*_2(1, 29)_ = 5.99, *p* = 0.02, η^2^_*p*_ = 0.06, as well as a significant main effect of the noun probed, *F*_1(1, 68)_ = 12.73, *p* = 0.001, *F*_2(1, 29)_ = 19.18, *p* < 0.001, η^2^_*p*_ = 0.16. Despite the pattern of means replicating the cross-experiment interaction seen in Experiments 1A and 1B, there was not a significant interaction between the number of nouns in the sentence and the noun being probed, *F*_1(1,68)_ = 0.28, *p* = 0.60, *F*_2(1, 29)_ = 2.64, *p* = 0.12. Planned pairwise comparisons revealed a non-significant 46 ms effect of the number of nouns for the antecedents, *t*_1(68)_ = 1.35, *p* = 0.18, *t*_2(29)_ = 0.80, *p* = 0.43, but the 73 ms effect of the number of nouns for distractor probes, though not significant by subjects, *t*_1(68)_ = 1.73, *p* = 0.09, was significant by items, *t*_2(29)_ = 3.00, *p* = 0.005, *d* = 0.21. For the sake of comparison with Experiments 1A and 1B, in Experiment 2A the slope of the number of nouns among the referents was 15.4 ms/noun, whereas the slope of the number of nouns among the distractors was 24.2 ms/noun. These values were 15.2 and 31.3, respectively, in Experiments 1A and 1B.

#### Reference-sentence reading times

Outliers were first identified as words read for less than 150 ms or more than 700 ms; different criteria were used in this experiment to try to approximate in a per-word measure the per-character measures used in the previous experiments. Outliers among the remaining reading times were then identified within each subject based on Tukey's (1977) criteria. This resulted in 8.1% of the trials being excluded from further analysis^3^.

The individual-word reading times were combined into three regions of three words each. The pre-anaphor region consisted of the three words prior to the anaphor; the anaphor region consisted of the three-word noun phrase involving the determiner, adjective, and anaphor (e.g., *the cutting tool*); and the post-anaphor region consisted of the three words following the anaphor. Although some items had more than three words prior to the anaphor noun phrase, the analysis was restricted to this point because there was a dramatic drop in the number of observations starting four words prior to the anaphor region. The post-anaphor region was always the final three words of the anaphor sentence. Thus, each region consisted of three words, making their reading times roughly comparable.

**Figure 3:**
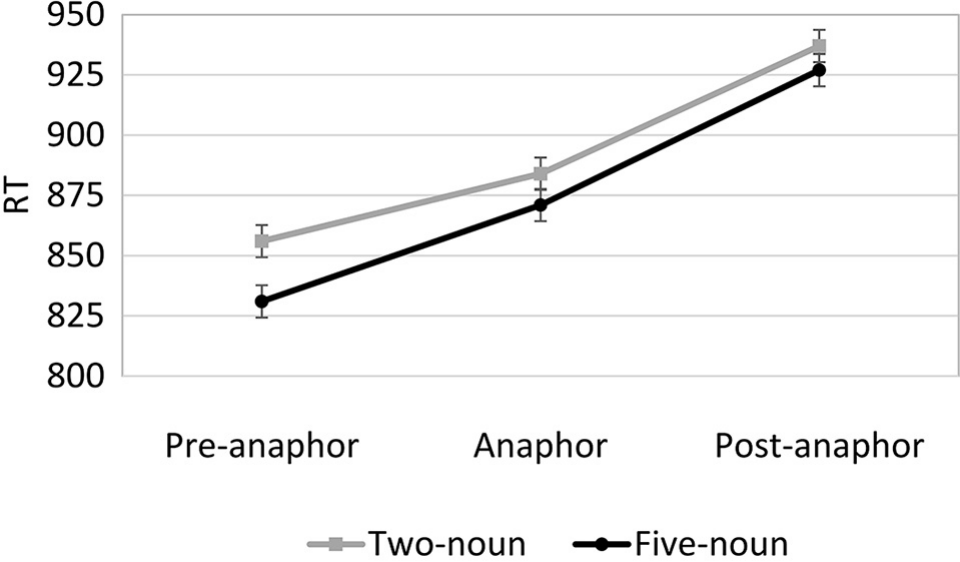
**Experiment 2A mean anaphor sentence reading times per region (error bars indicate SE of the mean)**.

In general, reading time on the reference sentence decreased as the number of nouns in the list sentence increased (see Figure 3); this effect occurred most strongly in the pre-anaphor region. A 2 (nouns: 2, 5) × 3 (region: pre-anaphor, anaphor, post-anaphor) repeated measures ANOVA revealed a significant main effect of the number of nouns only in the items analysis, *F*_1(2, 68)_ = 2.68, *p* = 0.11, *F*_2(1, 29)_ = 4.66, *p* = 0.04, η^2^_*p*_ = 0.04. There was also a significant main effect of region, *F*_1(2, 136)_ = 29.9, *p* < 0.001, *F*_2(1, 58)_ = 16.8, *p* < 0.001, η^2^_*p*_ = 0.31, but the interaction between the number of nouns and region was non-significant, *F*_1(2, 136)_ = 0.65, *p* = 0.53, *F*_2(1, 58)_ = 1.03, *p* = 0.36. Planned pairwise comparisons revealed that subjects read the pre-anaphor region significantly faster in the five noun condition compared to the two noun condition (*p* = 0.02 by subjects, *p* = 0.05 by items), but this effect was non-significant in the anaphor region (*p* =0.26 by subjects, *p* = 0.16 by items) and the post-anaphor region (*p* = 0.51 by subjects, *p* = 0.21 by items).

### Discussion

As predicted by the fan-effect hypothesis, and consistent with Experiments 1A and 1B, probe word accuracy was higher and responses were made faster in the two-noun condition than in the five-noun condition for both referents and distractors. Moreover, the cross-experiment interaction of number of nouns and probe type was replicated; the fan effect is larger for distractors. The reading time results replicated those from Experiments 1A and 1B: subjects read the reference sentence faster in the five-noun condition than in the two-noun condition. However, measuring reading time per-word enabled a more detailed analysis of the reference-sentence reading times and revealed that the faster reading primarily occurred in the pre-anaphor region. Because this region was identical across conditions and made no reference to the list sentence, there is no theoretical reason to expect this difference based on anaphor resolution processes. Instead, these results support the speeded-reading explanation suggested in the discussion of Experiment 1A, that subjects may have adopted a particular strategy in order to mitigate the increased difficulty of the probe-word task in the five-noun condition by reaching the probe word task and comprehension questions more quickly. Furthermore, per-character reading times on the list sentence (see Appendix B in Supplementary Material) increased as the number of nouns increased, suggesting that the speeded-reading strategy was adopted only on the reference sentence after subjects became aware of the increased difficulty imposed by the longer lists.

## Experiment 2B

Because subjects appeared to be adjusting their reading speed to accommodate the difficulty of representing multiple referents, it was important to assess whether the probe word results were dependent on this apparent strategy. Experiment 2B was thus a replication of Experiment 2A using a fixed-rate presentation of the passages. By controlling the pace of reading, any effects found on the probe recognition task can be assumed to reflect processes that occurred independent of subjects' variable reading speed. Holding reading-rate constant was not expected to change the probe-word results, so it was expected that responses to both referents and distractors would be faster and more accurate when there were two referents in the list sentence than when there were five referents. Moreover, this experiment provided one more opportunity to examine the prediction that the effect of fan would be greater among distractors than among referents. In the accuracy data, the fan effect has been reliably much stronger for distractors than it has been among referents. In the reaction-time data, between Experiments 1A and 1B, this effect was significant only in a one-tailed test, and in Experiment 2A, the same pattern emerged but it was not reliable.

### Method

#### Subjects

Sixty-six students enrolled in a general psychology course at the University of Arkansas participated in the experiment to partially fulfill a research requirement. All subjects were native-English speakers.

#### Materials, design, and procedure

The materials, design, and procedure were identical to Experiment 2A except that the materials were presented at a fixed pace of 450 ms per word^4^.

### Results

#### Data exclusion and general analytic considerations

Based on the data exclusion criteria, the data from 10 subjects were excluded from further analysis. Four more subjects were removed from further analysis for a systematic misunderstanding of the instructions. Therefore, the analyses included 50 subjects and 30 items.

#### Comprehension

In general, comprehension (see Table 2) decreased as the number of nouns increased. A 2 (nouns: 2, 5) × 2 (noun probed: referent, distractor) repeated-measures ANOVA revealed a main effect of nouns that was significant in the subject analysis, *F*_1(1, 49)_ = 4.59, *p* = 0.04, η^2^_*p*_ = 0.09, but non-significant in the items analysis, *F*_2(1, 29)_ = 2.53, *p* = 0.12. The main effect of noun probed was non-significant, *F*_1(1, 49)_ = 0.20, *p* = 0.66, *F*_2(1, 29)_ = 0.23, *p* = 0.63, and the interaction between number of nouns and noun probed was also non-significant, *F*_1(1, 49)_ = 1.50, *p* = 0.23, *F*_2(1, 29)_ = 1.31, *p* = 0.26.

#### Probe accuracy

Table 5 presents mean accuracy and probe reaction times as a function of the number of nouns and the noun probed. In general, accuracy was higher for referents than for distractors and when there were two nouns in the list sentence than when there were five, once again replicating the pattern seen in Experiments 1A, 1B, and 2A. A 2 (nouns: 2, 5) × 2 (noun probed: referent, distractor) repeated-measures ANOVA revealed a significant main effect of the number of nouns, *F*_1(1, 49)_ = 53.9, *p* < 0.001, *F*_2(1, 29)_ = 27.9, *p* < 0.001, η^2^_*p*_ = 0.52, as well as a significant main effect of the noun probed, *F*_1(1, 49)_ = 53.0, *p* < 0.001, *F*_2(1, 29)_ = 31.8, *p* < 0.001, η^2^_*p*_ = 0.52. Additionally, there was a significant interaction between the number of nouns in the sentence and the noun being probed, *F*_1(1, 49)_ = 29.2, *p* < 0.001, *F*_2(1, 29)_ = 23.1, *p* < 0.001, η^2^_*p*_ = 0.37, with a greater 2- vs. 5-noun difference for distractors than for referents, the third time this pattern has been replicated.

#### Probe reaction times

Based on outlier exclusion criteria, 8.7% of the data were excluded from further analyses. Like the accuracy results, reaction time tended to be shorter for referents than for distractors and when there were two nouns in the list sentence than when there were five. A 2 (nouns: 2, 5) × 2 (noun probed: referent, distractor) repeated-measures ANOVA showed that there was a significant main effect of the number of nouns, *F*_1(1, 49)_ = 25.8, *p* < 0.001, *F*_2(1, 29)_ = 19.4, *p* < 0.001, η^2^_*p*_ = 0.35, as well as a significant main effect of the noun probed, *F*_1(1, 49)_ = 44.1, *p* < 0.001, *F*_2(1, 29)_ = 31.9, *p* < 0.001, η^2^_*p*_ = 0.47. Once again, the pattern of means replicated the cross-experiment pattern seen in Experiments 1A and 1B as well as that seen in Experiment 2A, with the effect of number of nouns being larger for distractors than for referents. Despite this, there was not a significant interaction between the number of nouns in the sentence and the noun being probed, *F*_1(1, 49)_ = 0.70, *p* = 0.41, *F*_2(1, 29)_ = 2.03, *p* = 0.17. The effect of the number of nouns was significant among the referents, *t*_1(49)_ = 2.86, *p* = 0.006, *t*_2(29)_ = 2.54, *p* = 0.02, as well as among the distractors, *t*_1(49)_ = 4.55, *p* < 0.001, *t*_2(29)_ = 4.87, *p* < 0.001; this effect was numerically smaller for referents (94 ms, *d* = 0.40) than for distractors (130 ms, *d* = 0.64). The slopes corresponding to these effects, 31.2 ms/noun for referents and 43.2 ms/noun for the distractors, were substantially larger than the respective slopes seen in the previous experiments, possibly due to the change in the presentation of the passages to experimenter-paced.

### Discussion

The results confirmed the predictions of the fan-effect hypothesis, and the probe-word results were conceptually identical to Experiment 2A. Although subjects in the previous experiments seemed to be adopting a special strategy of reading the reference sentence more quickly when there were more distractors, the results of Experiment 2B indicate that this strategy was not necessary for the emergence of the probe-word results we had previously observed because subjects did not have the opportunity to employ it. The replication of the finding that the activation level of nouns decreases as the number of distractors increases therefore appears to be the result of a diffusion of activation to all potential referents.

However, this conclusion relies on the assumption that subjects were resolving the anaphor and that the anaphor processing affected the activation level of the referents. There is some evidence, however, that anaphor resolution may not always occur during reading (Greene et al., 1992; Levine et al., 2000; Klin et al., 2004, 2006; Love and McKoon, 2011), making it possible that the present results could be occurring independent of anaphor resolution. The effect of nouns may have been caused by the increasing memory demands incurred as the number of referents increased regardless of whether the subjects attempted to resolve the anaphors. It is possible that as the amount of information in the subjects' mental representations increased, the probability of the correct referent being activated even by the probe word itself, independent of anaphor resolution processes, decreased, resulting in slower reaction times as the number of referents.

## Experiment 3

Experiment 3 was designed to address the possibility that anaphors were not being resolved in the prior experiments. To do so, the reference sentence was modified such that it contained an anaphor or not (see Table 1), a manipulation that has been used many times in the anaphor resolution literature (e.g., Dell et al., 1983; Levine et al., 2000). As in Experiments 2A and 2B, there were either two nouns (i.e., a referent and one distractor) or five nouns (i.e., a referent and four distractors) in the list sentence that preceded the reference sentence. The referent was used as the probe word to provide an index of the activation of this concept at the end of the anaphor or no-anaphor sentence. According to the fan-effect hypothesis, it is activation from the anaphor as a memory cue that is divided among the referent and the distractors that is the source of the effect of the number of nouns. Thus, when there is an anaphor, the fan-effect hypothesis predicts an effect of the number of nouns like that seen in the previous experiments. Whatever pattern emerges for the effect of the number of nouns in the anaphor condition, because anaphor resolution involves reactivation of the correct referent (e.g., Dell et al., 1983), there should be an overall accuracy and reaction time advantage in the anaphor over the no-anaphor control condition.

### Method

#### Subjects

Seventy students enrolled in a general psychology course at the University of Arkansas participated in the experiment to partially fulfill a research requirement. All subjects were native-English speakers.

#### Materials and design

Experiment 3 used the same set of materials as Experiments 2A and 2B with the exception that the reference sentence was manipulated (see Table 1) such that it included an anaphor (i.e., Anaphor condition) or not (i.e., No Anaphor condition), while equating for length (i.e., the mean length for both the anaphor and no anaphor conditions was 61.5 characters). Finally, the probe words were limited to referents only, as in Experiment 1A. The manipulation of these factors resulted in a 2 (nouns: 2, 5) × 2 (reference: anaphor, no anaphor) completely within-subjects design.

#### Procedure

The procedure of Experiment 3 was identical to that of Experiments 1A and 1B, except that it included only 98 trials (30 experimental and 68 fillers), as in Experiments 2A and 2B.

### Results

#### Data exclusion and general analytic considerations

Based on outlier identification and comprehension and probe accuracy, the data from 5 subjects were excluded from further analysis. Therefore, the reported analyses included 65 subjects and 30 items.

#### Comprehension

In general, comprehension (see Table 2) decreased as the number of nouns increased and accuracy was greater in the anaphor condition than in the no anaphor condition. A 2 (nouns: 2, 5) × 2 (reference: anaphor, no anaphor) repeated measures ANOVA revealed a non-significant main effect of nouns, *F*_1(1, 64)_ = 0.62, *p* = 0.44, *F*_2(1, 30)_ = 0.90, *p* = 0.35, and a significant main effect of reference, *F*_1(1, 64)_ = 9.28, *p* = 0.003, *F*_2(1, 30)_ = 6.15, *p* = 0.019, η^2^_*p*_ = 0.13. In addition, there was a significant interaction between the number of nouns and reference, *F*_1(1, 64)_ = 4.83, *p* = 0.032, *F*_2(1, 30)_ = 5.23, *p* = 0.029, η^2^_*p*_ = 0.07, with a 7.3% accuracy advantage for the 2-noun condition compared to the 5-noun condition in the anaphor condition but only a 1.3% accuracy advantage in the no anaphor condition. However, the comprehension questions differed between the anaphor and no anaphor conditions, making this the likely cause of the observed effect.

#### Probe accuracy

Table 6 presents mean probe word accuracy and reaction times along with mean reference-sentence reading times as a function of the number of referents and whether the reference sentence contained an anaphor. In general, subjects responded more accurately in the anaphor condition than in the no anaphor condition and when there were two nouns in the list sentence than when there were five nouns. A 2 (nouns: 2, 5) × 2 (reference: anaphor, no anaphor) repeated measures ANOVA revealed a significant main effect of the number of nouns, *F*_1(1, 64)_ = 8.43, *p* = 0.005, *F*_2(1, 29)_ = 14.14, *p* = 0.001, η^2^_*p*_ = 0.12; however, the simple effect of the number of nouns for the anaphor condition was not significant, *t*_1(64)_ = 1.11, *p* = 0.27, *t*_2(29)_ = 1.21, *p* = 0.24. There was also a significant main effect of reference, *F*_1(1, 64)_ = 7.53, *p* = 0.008, *F*_2(1, 29)_ = 6.56, *p* = 0.02, η^2^_*p*_ = 0.11, but the interaction between the number of nouns and reference was non-significant, *F*_1(1, 64)_ = 2.70, *p* = 0.11, *F*_2(1, 29)_ = 2.84, *p* = 0.10.

**Table 6:** **Experiment 3 mean probe word responses and per-character reading times (with standard errors of the mean)**.

	**Anaphor condition**			
	**Probe word responses**		**Per-character reading time**	
	**Accuracy**	**Reaction time**	**List sentence**	**Reference sentence**
Two-noun	0.96 (0.01)	976 (9.9)	70.7 (1.32)	55.7 (0.64)
Five-noun	0.94 (0.01)	997 (9.9)	73.7 (1.32)	54.0 (0.64)
	**No anaphor condition**			
	**Probe word responses**		**Per-character reading time**	
	**Accuracy**	**Reaction time**	**List sentence**	**Reference sentence**
Two-noun	0.95 (0.01)	1009 (9.9)	–	51.2 (0.64)
Five-noun	0.89 (0.01)	1013 (9.9)	–	50.1 (0.64)

#### Probe reaction times

Based on outlier exclusion criteria, 7.5% of the data were excluded from further analyses. Reaction times (see Table 6) tended to be faster in the anaphor condition than in the no anaphor condition and when there were two nouns in the list sentence than when there were five nouns. A 2 (nouns: 2, 5) × 2 (reference: anaphor, no anaphor) repeated measures ANOVA revealed that the main effect of nouns was non-significant, *F*_1(1, 64)_ = 1.26, *p* = 0.27, *F*_2(1, 29)_ = 0.24, *p* = 0.63. Because of the prediction of the fan-effect hypothesis, the effect of the number of nouns was examined for the anaphor condition. The noun-effect was 23 ms but was also not significant, *t*_1(64)_ = 1.26, *p* = 0.21, *t*_2(29)_ = 0.80, *p* = 0.43. The main effect of reference was nearly significant in the subjects analysis, *F*_1(1, 64)_ = 3.38, *p* = 0.07, η^2^_*p*_ = 0.05, but non-significant in the items analysis, *F*_2(1, 29)_ = 2.32, *p* = 0.14, and the interaction between reference and nouns was not significant, *F*_1(1, 64)_ = 0.65, *p* = 0.42, *F*_2(1, 29)_ = 1.09, *p* = 0.31.

#### Reference-sentence reading times

Based on outlier exclusion criteria, 6.5% of the data were excluded from further analyses. In general, reading time (see Table 6) was longer when the sentence contained an anaphor than when it did not. A 2 (nouns: 2, 5) × 2 (reference: anaphor, no anaphor) repeated measures ANOVA revealed a significant main effect of reference, *F*_1(1, 64)_ = 23.29, *p* < 0.001, *F*_2(1, 29)_ = 13.01, *p* = 0.001, η^2^_*p*_ = 0.27. The main effect of nouns was not quite significant, *F*_1(1, 64)_ = 3.08, *p* = 0.08, *F*_2(1, 29)_ = 1.56, *p* = 0.22, although the pattern observed in Experiments 1A, 1B, and 2A appeared once again, with shorter reading times when there were more nouns. There was not a significant interaction between reference and nouns, *F*_1(1, 64)_ = 0.23, *p* = 0.64, *F*_2(1, 29)_ = 0.55, *p* = 0.46.

### Discussion

The results from Experiment 3 provided some evidence that subjects were in fact resolving the anaphors when reading the passages. Probe accuracy was better after reading a sentence with an anaphoric reference than after reading a sentence that did not make an anaphoric reference. Additional evidence that subjects were resolving the anaphors comes from the reading-time data. Controlling for length, the reference sentences were read more slowly when they contained an anaphor than when they did not, consistent with the hypothesis that subjects were engaging in additional processing to resolve the anaphor. This conclusion is tentative, though, as there were more explicit references^5^ (e.g., pronouns, specifiers, definite noun phrases) to entities in the prior sentence in the reference sentences in the anaphor condition (*M* = 2.8, *SD* = 0.8) than in the no-anaphor condition (*M* = 1.6, *SD* = 0.8), *t*_(29)_ = 6.27, *p* < 0.001. In most cases (25 of 30 passages), these additional references were not to any of the list items; excluding the five passages with a second reference to list items does not change the pattern of results for probe accuracy or reaction time reported above.

The results of this experiment's anaphor condition were less consistent with the fan-effect hypothesis than the results from prior experiments, although the general pattern of degraded recognition performance with more nouns persisted; we return to this issue in the General Discussion.

## General discussion

Explanations of how anaphoric expressions are understood have frequently appealed to general memory processes. Consistent with theories of comprehension that place memory at their center (e.g., Kintsch, 1988; Myers and O'Brien, 1998; Lewis and Vasishth, 2005), anaphor resolution is more difficult when factors are present that make retrieving a unique item from memory more difficult, such as when there is similarity between a desired target and some distractor. Prior research that has produced findings that are consistent with this hypothesis (e.g., Corbett and Chang, 1983; Corbett, 1984; O'Brien, 1987; O'Brien et al., 1990; Greene et al., 1992; Levine et al., 2000; Badecker and Straub, 2002; Klin et al., 2004, 2006) have used stimuli with one distractor and one antecedent, and by a variety of measures anaphor resolution has been shown to be more difficult because of the distractor. In five experiments, we examined the hypothesis that a greater number of distractors would lead to a fan effect (Anderson, 1974) in anaphor resolution, that is, if with each additional distractor there would be additional difficulty in identifying the correct referent of the anaphor. We also examined the effect of additional distractors on the activation of those distractors. Our subjects read pairs of sentences, the first of which provided a variably-long list of concepts from the same taxonomic category and the second of which made unambiguous reference to one of the items in the list with an adjective-modified definite noun phrase; this was followed by a probe recognition task that should provide an index of how active the probed concept is in the text representation.

Collectively, the probe word results from the present experiments supported the hypothesis that distractors have a cumulative effect on antecedent activation levels. Although the effect of the distractors on reaction time varied in size and significance from experiment to experiment, it is overall a robust effect. The two- and five-noun conditions with a referent probe were present in Experiments 1A, 2A, 2B, and 3. The subject data from these four experiments were combined and submitted to a 2 (nouns: 2, 5) × 4 (Experiments: 1A, 2A, 2B, 3) mixed-factor ANOVA with repeated-measures on the first factor. The effect of nouns was significant, *F*_(1, 265)_ = 15.56, *p* < 0.001, and the interaction was not, *F*_(3, 265)_ = 1.18, *p* = 0.32, suggesting that there was not significant variability in the effect of nouns across experiments. Cohen's *d* for the effect of nouns was 0.24 (95% confidence interval: 0.12, 0.36; Smithson, 2003), demonstrating a small but reliable effect. Whereas previous research has shown that the presence of a single distractor interferes with the activation of the antecedent, the present research extends this finding by demonstrating that each additional distractor further reduces the activation level of the antecedent and other distractors. This effect is akin to a set size effect (Sternberg, 1966), with larger lists leading to longer reaction times; however, the difference in the size of the effect for referents and distractors suggests that an additional process related to anaphor resolution is also occurring.

The present results are conceptually similar to the fan effect where delayed recognition [i.e., the recognition task occurring after the presentation of all of the materials as in Anderson (1974)] slows as the number of facts associated with a noun increases. This effect is generally attributed to the reduction in the probability of the correct item in memory being activated at the time of retrieval, thus slowing responses. The present experiments demonstrate an earlier effect, with the number of distractors affecting the activation level of nouns immediately after each trial. In this case, the categorical anaphor (e.g., *tool*) acts as a retrieval cue, with activation being split among all of the concepts associated with the category (i.e., the referent and distractor[s]). Increasing the number of distractors should therefore increase the time required to resolve the anaphor. This increased retrieval time effect was not observed in the present experiments, although this was likely due to subjects adopting a speeded-reading strategy (see the discussion of the reading-time results below). As a consequence of multiple potential antecedents, activation should be divided among the concepts, limiting the activation for each one (see spreading activation theory; Collins and Loftus, 1975; Anderson, 1983). This prediction was supported by the slowed reaction times and the reduced accuracy resulting from increasing the number of distractors. The present results further demonstrate that activation does not spread equally to all category members when there is disambiguating information (e.g., an adjective modifier like *cutting* in *the cutting tool*). Increasing the number of nouns led to a consistently greater reduction in probe accuracy and increase in reaction time for distractors than for referents in the Experiments 1A and 1B combined analysis and Experiments 2A and 2B, suggesting that activation was spreading disproportionately to the referent.

We have framed the current results as primarily being an effect that occurs at the time of retrieval (i.e., upon reading the anaphor). It is possible that these effects are also influenced by encoding or storage interference. Upon reading multiple items with many shared features, like our list-sentence items, the mental representation of these items may be overwritten (Nairne, 1990) or degraded due to repeated reactivation by similar items (Estes, 1997). The methodology used in the current research does not allow for delineation between a storage-based and a retrieval-based explanation. Ferreting out the relative contributions of storage- and retrieval-interference processes would likely require careful parametric manipulation of feature overlap among distractors and the referent as well as precise control over not only timing of reading and probes but also time elapsed between storage and retrieval, as well as manipulation of serial position of distractors and referents. Attempting to work out these details is a promising avenue for future research.

Turning to the reading-time results, we found no evidence that additional distractors led to more difficulty processing anaphoric reference. By contrast, we consistently found that our subjects read faster as there were more distractors. We believe that this is the result of subjects adopting a speeded-reading strategy on difficult trials (i.e., trials with longer lists of nouns), which counteracted the predicted increase in anaphor reading time. This is similar to Van Dyke and McElree's (2006) finding that, while reading grammatically-complex sentences, subjects read faster and had worse comprehension while holding a memory load (i.e., a list of three words) than when not holding a memory load, suggesting a dual-task strategic trade-off. Our subjects also had lower comprehension with greater list length (see Table 2), suggesting that there was possibly a task demand that shifted attention somewhat from the comprehension aspect of the task to the memory aspect of the task. In no case, however, was comprehension lower than about 83%. Moreover, there is no theoretical reason to expect anaphor resolution to take less time as the number of candidate antecedents increases unless subjects were giving up on trying to identify the correct antecedent (Levine et al., 2000). There are a few arguments consistent with the notion that subjects were in fact resolving the anaphors in the current research. First, correctly answering a large majority of the comprehension questions required the anaphors to be resolved, which some have suggested is necessary to get subjects to resolve anaphors in anaphor resolution research (Foertsch and Gernsbacher, 1994). Second, some subjects, especially in Experiment 1B, spontaneously adopted the strategy of labeling distractors as new in the probe recognition task, which suggests that they had selected the referent as the “correct” answer and distractors as the “incorrect” answer to the probe task. Third, Experiment 3 provides tentative evidence that subjects were resolving the anaphor, even on five-noun trials. Given these arguments and findings, we believe that our subjects were resolving anaphors even when it was difficult to do so. Therefore, the speeded-reading strategy appears to be the most parsimonious explanation of these unexpected results. Furthermore, the fixed-pace presentation of the sentence in Experiment 2B prevented subjects from engaging in the speeded-reading strategy, demonstrating that the probe word effects do not rely on such a strategy. Future research should attempt to prevent the speeded-reading strategy while maintaining naturalistic reading (e.g., introducing a substantial delay between the passages and the probe task or eliminating the probe task entirely) in order to better evaluate the anaphor reading time hypothesis.

Finally, returning to the fan-effect hypothesis, the original explanation offered for the fan effect by Anderson (1974) was based on Anderson and Bower's (1973) theory of memory, which assumed that memory retrieval was based on search cues being used to identify, in parallel, matching elements in memory, which were then serially examined, resulting in an increase in reaction time with each additional matching element. In the former detail (i.e., a parallel matching), this theory is in the same family as other global-matching memory theories like those of Ratcliff (1978), Gillund and Shiffrin (1984), and Hintzman (1986), upon which memory-based text processing frameworks like Myers and O'Brien's (1998) resonance model are based. In this sense, the results of our experiments are confirmation of both theories of memory search and the hypothesis that at least some aspects of comprehension may be explained by general memory processes. However, other research into the fan effect has shown that there are circumstances under which there is no fan effect despite there being multiple associations with a single memory cue (Myers et al., 1984; Radvansky, 1998; Radvansky et al., 1998). Myers et al. found no fan effect when memory elements could be integrated causally. For example, reading the elements *the doctor went to the racetrack*, *the doctor studied the odds*, and *the doctor made a selection* may be readily integrated into a causally-coherent narrative representation about events occurring at a racetrack. Similarly, Radvansky and colleagues showed that the fan effect is reduced or even eliminated when potentially-competing memory elements can be readily integrated. One feature that makes elements easy to integrate is if they can occur at the same time (e.g., the grocer was folding a towel; the grocer was clearing his throat), whereas elements that are in different locations may not be integrated (e.g., the welcome mat is in the cocktail lounge; the welcome mat is in the office building). Radvansky et al. observed a fan-effect in recognition of hard-to-integrate elements, but not for easy-to-integrate elements. Given that there are boundary conditions for the fan-effect in memory experiments, a natural question to ask is if there are circumstances under which the search process in anaphor resolution might occur without interference. Across sentences, one such circumstance might be if the items in a list occur in more-naturalistic texts, allowing for an integrated situation model to be constructed, as suggested by both Myers et al. (1984) and Radvansky (1998; Radvansky et al., 1998). By contrast, within sentences, one condition that has been shown to limit the search for referents is when there are strong grammatical constraints on reference. Recent evidence from Dillon et al. (2013; see also Chow et al., 2014) suggests that syntactic principles may guide retrieval in a constrained manner for some linguistic dependencies, such as reflexives (but see Badecker and Straub, 2002; Kennison, 2003 and Sturt, 2003, for further complexities), leading to retrieval without interference from distractors; syntactic constraints may play an especially critical role in directing the retrieval processes that occur within a sentence. These types of findings are representative of two distinct research literatures have arisen over the past few decades, one focused on retrieval across sentences, and the other focused on retrieval within sentences. Integration of these theories and findings holds out the promise of yet further integration of theories of memory and comprehension.

## Author note

Portions of these data were presented at the 52nd Annual Meeting of the Psychonomic Society, 2011, Seattle, Washington, and at the 22nd Annual Meeting of the Society for Text and Discourse, 2012, Montreal, Canada.

### Conflict of interest statement

The authors declare that the research was conducted in the absence of any commercial or financial relationships that could be construed as a potential conflict of interest.

## Footnotes

^1^Experimenter error resulted in an odd number of experimental items in this experiment and in Experiment 1b.

^2^Probe length and frequency was similar for the two sets of materials (length: Set 1 *M* = 6.5 letters, *SD* = 1.5; Set 2 *M* = 6.4 letters, *SD* = 1.7; log frequency (Lund and Burgess, 1996; Balota et al., 2007): Set 1 *M* = 7.4, *SD* = 2.4; Set 2 *M* = 7.2, *SD* = 2.9).

^3^The pattern of results remained similar using a less-strict cutoff of 1500 ms/word.

^4^Due to limitations of the Linger program, words were presented at a fixed rate instead of using a variable rate dependent on the length of each word (cf. Gernsbacher, 1989).

^5^Associative anaphora (e.g., referring to *the test* after a sentence mentioning studying) were not counted.

## References

[B1] Almor A. (1999). Noun-phrase anaphora and focus: the informational load hypothesis. Psychol. Rev. 106, 748–765 10.1037/0033-295X.106.4.748 10560327

[B3] Anderson J. R. (1974). Retrieval of propositional information from long-term memory. Cogn. Psychol. 6, 451–474 10.1016/0010-0285(74)90021-8

[B4] Anderson J. R. (1983). A spreading activation theory of memory. J. Verbal Learn. Verbal Behav. 22, 251–295 10.1016/S0022-5371(83)90201-3

[B5] Anderson J. R. (2005). Human symbol manipulation within an integrated cognitive architecture. Cogn. Sci. 29, 313–341 10.1207/s15516709cog0000_22 21702777

[B6] Anderson J. R. Bower G. H. (1973). Human Associative Memory. Washington, DC: Winston and Sons

[B7] Anderson J. R. Reder L. M. (1999). The fan effect: new results and new theories. J. Exp. Psychol. Gen. 128, 186–197 10.1037/0096-3445.128.2.18617196744

[B8] Badecker W. Straub K. (2002). The processing role of structural constraints on the interpretation of pronouns and anaphors. J. Exp. Psychol. Learn. Mem. Cogn. 28, 748–769 10.1037/0278-7393.28.4.748 12109766

[B9] Balota D. A. Yap M. J. Cortese M. J. Hutchison K. A. Kessler B. Loftis B. (2007). The English lexicon project. Behav. Res. Methods 39, 445–459 10.3758/BF03193014 17958156

[B10] Chambers C. G. Smyth R. (1998). Structural parallelism and discourse coherence: a test of centering theory. J. Mem. Lang. 39, 593–608 10.1006/jmla.1998.2575

[B11] Chow W.-Y. Lewis S. Phillips C. (2014). Immediate sensitivity to structural constraints in pronoun resolution. Front. Psychol. 5:630 10.3389/fpsyg.2014.00630 25018739 PMC4073625

[B12] Collins A. M. Loftus E. F. (1975). A spreading-activation theory of semantic processing. Psychol. Rev. 82, 407–428 10.1037/0033-295X.82.6.407

[B13] Corbett A. T. (1984). Prenominal adjectives and the disambiguation of anaphoric nouns. J. Verbal Learn. Verbal Behav. 23, 683–695 10.1016/S0022-5371(84)90418-3

[B14] Corbett A. T. Chang F. R. (1983). Pronoun disambiguation: accessing potential antecedents. Mem. Cognit. 11, 283–294 10.3758/BF03196975 6621344

[B15] Dell G. S. McKoon G. Ratcliff R. (1983). The activation of antecedent information during the processing of anaphoric reference in reading. J. Verbal Learn. Verbal Behav. 22, 121–132 10.1016/S0022-5371(83)80010-3

[B16] Dillon B. Mishler A. Sloggett S. Phillips C. (2013). Contrasting intrusion profiles for agreement and anaphora: experimental and modeling evidence. J. Mem. Lang. 69, 85–103 10.1016/j.jml.2013.04.003

[B17] Ditman T. Holcomb P. J. Kuperberg G. R. (2007). The contributions of lexico-semantic and discourse information to the resolution of ambiguous categorical anaphors. Lang. Cogn. Process. 22, 793–827 10.1080/01690960601057126

[B18] Estes W. K. (1997). Processes of memory loss, recovery, and distortion. Psychol. Rev. 104, 148–169 10.1037/0033-295X.104.1.148 9009883

[B19] Foertsch J. Gernsbacher M. A. (1994). In search of comprehension: getting “minimalists” to work. Discourse Process. 18, 271–296 10.1080/01638539409544896 PMC4266472 25520530

[B20] Foraker S. McElree B. (2007). The role of prominence in pronoun resolution: active versus passive representations. J. Mem. Lang. 56, 357–383 10.1016/j.jml.2006.07.004

[B21] Garrod S. Sanford A. J. (1981). Bridging inferences and the extended domain of reference, in Attention and Performance IX, eds Long J. Baddeley A. (Hillsdale, NJ: Erlbaum), 331–346

[B22] Gernsbacher M. A. (1989). Mechanisms that improve referential access. Cognition 32, 99–156 10.1016/0010-0277(89)90001-2 2752708 PMC4467536

[B23] Gerrig R. McKoon G. (1998). The readiness is all: the functionality of memory-based text processing. Discourse Process. 26, 67–86 10.1080/01638539809545039

[B24] Gillund G. Shiffrin R. M. (1984). A retrieval model for both recognition and recall. Psychol. Rev. 91, 1–67 10.1037/0033-295X.91.1.1 6571421

[B26] Greene S. B. Gerrig R. J. McKoon G. Ratcliff R. (1994). Unheralded pronouns and management by common ground. J. Mem. Lang. 33, 511–526 10.1006/jmla.1994.1024

[B27] Greene S. B. McKoon G. Ratcliff R. (1992). Pronoun resolution and discourse models. J. Exp. Psychol. Learn. Mem. Cogn. 18, 266–283 10.1037/0278-7393.18.2.266 1532820

[B28] Hintzman D. L. (1986). “Schema abstraction” in a multiple-trace memory model. Psychol. Rev. 93, 411–428 10.1037/0033-295X.93.4.41122686158

[B29] Horton W. S. Gerrig R. J. (2005). Conversational common ground and memory processes in language production. Discourse Process. 40, 1–35 10.1207/s15326950dp4001_1

[B30] Just M. A. Carpenter P. A. Woolley J. D. (1982). Paradigms and processing in reading comprehension. J. Exp. Psychol. Gen. 111, 228–238 10.1037/0096-3445.111.2.228 6213735

[B31] Kennison S. M. (2003). Comprehending the pronouns *her, him*, and *his*: implications for theories of referential processing. J. Mem. Lang. 49, 335–352 10.1016/S0749-596X(03)00071-8

[B32] Kintsch W. (1988). The role of knowledge in discourse comprehension: a construction-integration model. Psychol. Rev. 95, 163–182 10.1037/0033-295X.95.2.163 3375398

[B34] Klin C. M. Guzmán A. E. Weingartner K. M. Ralano A. S. (2006). When anaphor resolution fails: partial encoding of anaphoric inferences. J. Mem. Lang. 54, 131–143 10.1016/j.jml.2005.09.001

[B35] Klin C. M. Weingartner K. M. Guzman A. E. Levine W. H. (2004). Readers' sensitivity to linguistic cues in narratives: how salience influences anaphor resolution. Mem. Cognit. 32, 511–522 10.3758/BF03195843 15285133

[B36] Levine W. H. Guzmán A. E. Klin C. M. (2000). When anaphor resolution fails. J. Mem. Lang. 43, 594–617 10.1006/jmla.2000.271921104564

[B37] Levine W. H. Hagaman J. A. (2008). Negated concepts interfere with anaphor resolution. Intercult. Pragmatics 5, 471–500 10.1515/IPRG.2008.023

[B38] Lewis R. L. (1996). Interference in short-term memory: the magical number two (or three) in sentence processing. J. Psycholinguist. Res. 25, 93–113 10.1007/BF01708421 8789368

[B39] Lewis R. L. Vasishth S. (2005). An activation-based model of sentence processing as skilled memory retrieval. Cogn. Sci. 29, 375–419 10.1207/s15516709cog0000_25 21702779

[B40] Loftus G. R. Masson M. E. J. (1994). Using confidence intervals in within-subject designs. Psychono. Bull. Rev. 1, 476–490 10.3758/BF03210951 24203555

[B40a] Lorch R. F. Myers J. L. (1990). Regression analyses of repeated measures data in cognitive research. J. Exp. Psychol. Learn. Mem. Cogn. 16, 149–157 10.1037/0278-7393.16.1.149 2136750

[B41] Love J. McKoon G. (2011). Rules of engagement: Incomplete and complete pronoun resolution. J. Exp. Psychol. Learn. Mem. Cogn. 37, 874–887 10.1037/a0022932 21480757 PMC3130815

[B42] Lund K. Burgess C. (1996). Producing high-dimensional semantic spaces from lexical co-occurrence. Behav. Res. Methods Instrum. Comput. 28, 203–208 10.3758/BF03204766

[B43] Mason R. A. (1997). The Role of Multiple Antecedents in the Time Course of Anaphor Resolution. Unpublished Master's thesis, University of Massachusetts, Amherst.

[B44] McElree B. (2000). Sentence comprehension is mediated by content-addressable memory structures. J. Psycholinguist. Res. 29, 111–123 10.1023/A:1005184709695 10709178

[B45] Myers J. L. O'Brien E. J. (1998). Accessing the discourse representation during reading. Discourse Process. 26, 131–157 21715401

[B46] Myers J. L. O'Brien E. J. Balota D. A. Toyofuku M. L. (1984). Memory search without interference: The role of integration. Cogn. Psychol. 16, 217–242 10.1016/0010-0285(84)90008-218515299

[B47] Nairne J. (1990). A feature model of immediate memory. Mem. Cognit. 18, 251–269 10.3758/BF03213879 2192233

[B48] O'Brien E. J. Plewes P. S. Albrecht J. E. (1990). Antecedent retrieval processes. J. Exp. Psychol. Learn. Mem. Cogn. 16, 241–249 2137863

[B49] O'Brien E. J. (1987). Antecedent search processes and the structure of text. J. Exp. Psychol. Learn. Mem. Cogn. 13, 278–290 10.1037/0278-7393.13.2.278 2952758

[B50] Radvansky G. A. (1998). The organization of information retrieved from situation models. Psychon. Bull. Rev. 5, 283–289 10.3758/BF03212952

[B51] Radvansky G. A. Zwaan R. A. Federico T. Franklin N. (1998). Retrieval from temporally organized situations models. J. Exp. Psychol. Learn. Mem. Cogn. 24, 1224–1237 10.1037/0278-7393.24.5.1224 9747531

[B52] Ratcliff R. (1978). A theory of memory retrieval. Psychol. Rev. 85, 59–108 10.1037/0033-295X.85.2.59

[B53] Reinhart T. (1983). Coreference and bound anaphora: a restatement of the anaphora questions. Linguist. Philos. 6, 47–88 10.1007/BF00868090

[B54] Rohde D. (2003). Linger: A Flexible Program for Language Processing Experiments. Available online at: http://tedlab.mit.edu/~dr/Linger/

[B55] Smithson M. (2003). Confidence Intervals: Quantitative Applications in the Social Sciences, *No. 140* Thousand Oaks, CA: Sage

[B56] Sternberg S. (1966). High-speed scanning in human memory. Science 153, 652–654 10.1126/science.153.3736.652 5939936

[B57] Sturt P. (2003). The time-course of the application of binding constraints in reference resolution. J. Mem. Lang. 48, 542–562 10.1016/S0749-596X(02)00536-3

[B58] Townsend J. T. Fifíc M. (2004). Parallel versus serial processing and individual differences in high-speed search in human memory. Percept. Psychophys. 66, 953–962 10.3758/BF03194987 15675643

[B62a] Tukey J. W. (1977). Exploratory Data Analysis. Reading, MA: Addison-Wesley

[B59] van den Broek P. Rapp D. N. Kendeou P. (2005). Integrating memory-based and constructionist processes in accounts of reading comprehension. Discourse Process. 39, 299–316 10.1080/0163853X.2005.9651685

[B60] Van Dyke J. A. McElree B. (2006). Retrieval interference in sentence comprehension. J. Mem. Lang. 55, 157–166 10.1016/j.jml.2006.03.007 18209744 PMC2206541

[B61] van Gompel R. P. G. Majid A. (2004). Antecedent frequency effects during the processing of pronouns. Cognition 90, 255–264 10.1016/S0010-0277(03)00161-6 14667697

[B62] Wiley J. Mason R. A. Myers J. L. (2001). Accessibility of potential referents following categorical anaphors. J. Exp. Psychol. Learn. Mem. Cogn. 27, 1238–1249 10.1037/0278-7393.27.5.1238 11550751

