# Supplementary material for: A fan effect in anaphor processing: effects of multiple distractors
Source: Front Psychol. 2014 Jul 29;5:818. doi: 10.3389/fpsyg.2014.00818 (PMC4114326; doi:10.3389/fpsyg.2014.00818)
Supplement: Supplementary file 1 [file DataSheet1.DOCX]

Appendix A

Experimental passages. Materials occurred in all experiments unless otherwise specified.

| List Sentence  2-noun  3-noun  4-noun  5-noun  Reference Sentence  Anaphor  No Anaphor  Probe Word  Referent  Distractor  Comprehension | Amelia’s new table was wobbling, so she looked in her toolbox and found …  a hammer and a saw.  a screwdriver, a hammer, and a saw. *(Experiments 1a and 1b only)*  a level, a screwdriver, a hammer, and a saw. *(Experiments 1a and 1b only)*  a wrench, a level, a screwdriver, a hammer, and a saw.  She fixed it with the cutting tool before it broke.  She fixed the table all by herself before it broke. *(Experiment 3 only)*  SAW *(Experiments 1a, 2a, 2a, and 2b)*  HAMMER *(Experiments 1a, 2a, and 2b)*  Did Amelia use the saw? |
| --- | --- |
| List Sentence  2-noun  3-noun  4-noun  5-noun  Reference Sentence  Anaphor  No Anaphor  Probe Word  Referent  Distractor  Comprehension | Isabella’s options for instruments she could play in the orchestra were …  the clarinet and violin.  the trombone, clarinet, and violin. *(Experiments 1a and 1b only)*  the trumpet, trombone, clarinet, and violin. *(Experiments 1a and 1b only)*  the tuba, trumpet, trombone, clarinet, and violin.  She chose the stringed instrument for its color.  She hoped she would be a famous musician someday. *(Experiment 3 only)*  VIOLIN *(Experiments 1a, 2a, 2a, and 2b)*  CLARINET *(Experiments 1a, 2a, and 2b)*  Did Isabella play the trombone? |
| List Sentence  2-noun  3-noun  4-noun  5-noun  Reference Sentence  Anaphor  No Anaphor  Probe Word  Referent  Distractor  Comprehension | Ava was restocking her kitchen with …  sugar and pepper.  salt, sugar, and pepper. *(Experiments 1a and 1b only)*  sage, salt, sugar, and pepper. *(Experiments 1a and 1b only)*  rosemary, sage, salt, sugar, and pepper.  That night, she cooked with the spicy ingredient in her apartment.  She couldn't remember the last time she had restocked her kitchen. *(Experiment 3 only)*  PEPPER *(Experiments 1a, 2a, 2a, and 2b)*  SUGAR *(Experiments 1a, 2a, and 2b)*  Did Ava cook with the sugar? |
| List Sentence  2-noun  3-noun  4-noun  5-noun  Reference Sentence  Anaphor  No Anaphor  Probe Word  Referent  Distractor  Comprehension | Noah planned to rob a bank, and for a weapon, he needed to choose between …  a machete and a grenade.  acid, a machete, and a grenade. *(Experiments 1a and 1b only)*  a pistol, acid, a machete, and a grenade. *(Experiments 1a and 1b only)*  a rifle, a pistol, acid, a machete, and a grenade.  He decided on the explosive weapon despite its size.  He needed the money to pay for his wife's operation. *(Experiment 3 only)*  GRENADE *(Experiments 1a, 2a, 2a, and 2b)*  MACHETE *(Experiments 1a, 2a, and 2b)*  Did Noah use the grenade? |
| List Sentence  2-noun  3-noun  4-noun  5-noun  Reference Sentence  Anaphor  No Anaphor  Probe Word  Referent  Distractor  Comprehension | Liam wanted to rent a boat for Labor Day weekend, and the marina had …  a rowboat and a yacht.  a skiff, a rowboat, and a yacht. *(Experiments 1a and 1b only)*  a houseboat, a skiff, a rowboat, and a yacht. *(Experiments 1a and 1b only)*  a barge, a houseboat, a skiff, a rowboat, and a yacht.  He rented the expensive boat from the attendant.  He loved any excuse to get the family together. *(Experiment 3 only)*  YACHT *(Experiments 1a, 2a, 2a, and 2b)*  ROWBOAT *(Experiments 1a, 2a, and 2b)*  Did Liam rent a rowboat? |
| List Sentence  2-noun  3-noun  4-noun  5-noun  Reference Sentence  Anaphor  No Anaphor  Probe Word  Referent  Distractor  Comprehension | At the fish market, Kaden saw …  swordfish and salmon.  trout, swordfish and salmon. *(Experiments 1a and 1b only)*  carp, trout, swordfish and salmon. *(Experiments 1a and 1b only)*  bass, carp, trout, swordfish and salmon.  For dinner he bought the pink fish wrapped in newspaper.  He liked to do his shopping on the weekends by himself. *(Experiment 3 only)*  SALMON *(Experiments 1a, 2a, 2a, and 2b)*  SWORDFISH *(Experiments 1a, 2a, and 2b)*  Did Kaden eat salmon? |
| List Sentence  2-noun  3-noun  4-noun  5-noun  Reference Sentence  Anaphor  No Anaphor  Probe Word  Referent  Distractor  Comprehension | Ethan’s schedule during the Fall semester included …  chemistry and calculus.  Spanish, chemistry, and calculus. *(Experiments 1a and 1b only)*  sociology, Spanish, chemistry, and calculus. *(Experiments 1a and 1b only)*  history, sociology, Spanish, chemistry, and calculus.  His worst grade was in the math class in early morning.  He had forever valued the importance of an education. *(Experiment 3 only)*  CALCULUS *(Experiments 1a, 2a, 2a, and 2b)*  CHEMISTRY *(Experiments 1a, 2a, and 2b)*  Did Ethan do poorly in chemistry? |
| List Sentence  2-noun  3-noun  4-noun  5-noun  Reference Sentence  Anaphor  No Anaphor  Probe Word  Referent  Distractor  Comprehension | The first day in her new house, Sophia saw …  a mosquito and a termite.  a fly, a mosquito, and a termite. *(Experiments 1a and 1b only)*  an ant, a fly, a mosquito, and a termite. *(Experiments 1a and 1b only)*  a roach, an ant, a fly, a mosquito, and a termite.  She called her boyfriend to come kill the wood-eating insect before she slept.  She told her boyfriend that night and he immediately called an exterminator. *(Experiment 3 only)*  TERMITE *(Experiments 1a, 2a, 2a, and 2b)*  MOSQUITO *(Experiments 1a, 2a, and 2b)*  Did Sophia call her boyfriend about the termite? |
| List Sentence  2-noun  3-noun  4-noun  5-noun  Reference Sentence  Anaphor  No Anaphor  Probe Word  Referent  Distractor  Comprehension | At the local jewelry shop, Olivia ordered a very expensive ring with …  a ruby and an emerald.  a pearl, a ruby, and an emerald. *(Experiments 1a and 1b only)*  a sapphire, a pearl, a ruby, and an emerald. *(Experiments 1a and 1b only)*  a diamond, a sapphire, a pearl, a ruby, and an emerald.  When it arrived, it was missing the green gem despite her instructions.  She was very wealthy and wanted to show everyone knew how rich she was. *(Experiment 3 only)*  EMERALD *(Experiments 1a, 2a, 2a, and 2b)*  RUBY *(Experiments 1a, 2a, and 2b)*  Was Olivia's ring onyx? |
| List Sentence  2-noun  3-noun  4-noun  5-noun  Reference Sentence  Anaphor  No Anaphor  Probe Word  Referent  Distractor  Comprehension | At the barbeque, Bree served …  sausage and pizza.  brisket, sausage, and pizza. *(Experiments 1a and 1b only)*  chicken, brisket, sausage, and pizza. *(Experiments 1a and 1b only)*  burgers, chicken, brisket, sausage, and pizza.  So many people came that she ran out of the cheesy dish before the end.  Unfortunately, she was cooking the whole time and didn't get to visit. *(Experiment 3 only)*  PIZZA *(Experiments 1a, 2a, 2a, and 2b)*  SAUSAGE *(Experiments 1a, 2a, and 2b)*  Did Bree serve pizza? |
| List Sentence  2-noun  3-noun  4-noun    5-noun  Reference Sentence  Anaphor    No Anaphor  Probe Word  Referent  Distractor  Comprehension | At the theater, Jackson ran the projector for …  a thriller and a comedy.  a mystery, a thriller, and a comedy. *(Experiments 1a and 1b only)*  a documentary, a mystery, a thriller, and a comedy. *(Experiments 1a and 1b only)*  a romance, a documentary, a mystery, a thriller, and a comedy.  When the weekend came, tickets were sold out for the funny movie despite its obscurity.  He was excited to have the weekend off from work and to spend time with his girlfriend. *(Experiment 3 only)*  COMEDY *(Experiments 1a, 2a, 2a, and 2b)*  THRILLER *(Experiments 1a, 2a, and 2b)*  Did the thriller sell out? |
| List Sentence  2-noun  3-noun  4-noun  5-noun  Reference Sentence  Anaphor    No Anaphor  Probe Word  Referent  Distractor  Comprehension | For his anatomy class, Landon studied …  the heart and the brain.  the appendix, the heart, and the brain. *(Experiments 1a and 1b only)*  the liver, the appendix, the heart, and the brain. *(Experiments 1a and 1b only)*  the stomach, the liver, the appendix, the heart, and the brain.  During the test, he remembered most about the gray organ without any difficulty.  He found it difficult to focus for more than thirty minutes without taking break. *(Experiment 3 only)*  BRAIN *(Experiments 1a, 2a, 2a, and 2b)*  HEART *(Experiments 1a, 2a, and 2b)*  Did Landon study the brain? |
| List Sentence  2-noun  3-noun  4-noun  5-noun  Reference Sentence  Anaphor  No Anaphor  Probe Word  Referent  Distractor  Comprehension | Jacob wanted to be a famous athlete, so he signed up for …  track and skiing.  baseball, track, and skiing. *(Experiments 1a and 1b only)*  soccer, baseball, track, and skiing. *(Experiments 1a and 1b only)*  football, soccer, baseball, track, and skiing.  In his senior year, he didn’t do the winter sport due to fatigue.  He found himself working out more than twice a day, on average. *(Experiment 3 only)*  SKIING *(Experiments 1a, 2a, 2a, and 2b)*  TRACK *(Experiments 1a, 2a, and 2b)*  Did Jacob ski? |
| List Sentence  2-noun  3-noun  4-noun  5-noun  Reference Sentence  Anaphor    No Anaphor  Probe Word  Referent  Distractor  Comprehension | At the animal shelter, Madeline adopted …  a mouse and a snake.  a bunny, a mouse, and a snake. *(Experiments 1a and 1b only)*  a cat, a bunny, a mouse, and a snake. *(Experiments 1a and 1b only)*  a dog, a cat, a bunny, a mouse, and a snake.  When she took them home, she forgot to name the reptilian animal before it died.  She had a very big heart and enjoyed every opportunity to offer a helping hand. *(Experiment 3 only)*  SNAKE *(Experiments 1a, 2a, 2a, and 2b)*  MOUSE *(Experiments 1a, 2a, and 2b)*  Did Madeline buy a ferret? |
| List Sentence  2-noun  3-noun  4-noun  5-noun  Reference Sentence  Anaphor  No Anaphor  Probe Word  Referent  Distractor  Comprehension | While studying for her test, Lily listened to …  metal and gospel.  rap, metal, and gospel. *(Experiments 1a and 1b only)*  rock, rap, metal, and gospel. *(Experiments 1a and 1b only)*  jazz, rock, rap, metal, and gospel.  She was distracted by the religious music loudly praising Jesus.  The silence always seemed to distract her more than anything else. *(Experiment 3 only)*  GOSPEL *(Experiments 1a, 2a, 2a, and 2b)*  METAL *(Experiments 1a, 2a, and 2b)*  Could Lily concentrate during gospel music? |
| List Sentence  2-noun  3-noun  4-noun  5-noun  Reference Sentence  Anaphor  No Anaphor  Probe Word  Referent  Distractor  Comprehension | Abigail’s garden needing some sprucing up, so she planted …  a rose and a sunflower.  an orchid, a rose, and a sunflower. *(Experiments 1a and 1b only)*  a daisy, an orchid, a rose, and a sunflower. *(Experiments 1a and 1b only)*  a lily, a daisy, an orchid, a rose, and a sunflower.  She was most excited about the tall flower in her garden.  She thought her home could use a little more character. *(Experiment 3 only)*  SUNFLOWER *(Experiments 1a, 2a, 2a, and 2b)*  ROSE *(Experiments 1a, 2a, and 2b)*  Did Abigail like sunflowers? |
| List Sentence  2-noun  3-noun  4-noun  5-noun  Reference Sentence  Anaphor  No Anaphor  Probe Word  Referent  Distractor  Comprehension | Braden’s many hobbies included …  sculpting and camping.  reading, sculpting, and camping. *(Experiments 1a and 1b only)*  juggling, reading, sculpting, and camping. *(Experiments 1a and 1b only)*  cooking, juggling, reading, sculpting, and camping.  He spent most of his time on the outdoors activity during the fall.  He was adventurous and always enjoyed learning about many new things. *(Experiment 3 only)*  CAMPING *(Experiments 1a, 2a, 2a, and 2b)*  SCULPTING *(Experiments 1a, 2a, and 2b)*  Did Braden hate camping? |
| List Sentence  2-noun  3-noun  4-noun  5-noun  Reference Sentence  Anaphor  No Anaphor  Probe Word  Referent  Distractor  Comprehension | Chloe’s new workout routine included …  yoga and swimming.  aerobics, yoga, and swimming. *(Experiments 1a and 1b only)*  biking, aerobics, yoga, and swimming. *(Experiments 1a and 1b only)*  jogging, biking, aerobics, yoga, and swimming.  She really liked the wet exercise at the beginning.  She was ready to lose a few pounds after the holidays. *(Experiment 3 only)*  SWIMMING *(Experiments 1a, 2a, 2a, and 2b)*  YOGA *(Experiments 1a, 2a, and 2b)*  Did Chloe prefer swimming? |
| List Sentence  2-noun  3-noun  4-noun  5-noun  Reference Sentence  Anaphor  No Anaphor  Probe Word  Referent  Distractor  Comprehension | At the diner, Emma often served …  juice and milk.  cocoa, juice, and milk. *(Experiments 1a and 1b only)*  coffee, cocoa, juice, and milk. *(Experiments 1a and 1b only)*  tea, coffee, cocoa, juice, and milk.  She personally did not like the dairy drink on her tongue.  She was beginning to get bored of her repetitive routine. *(Experiment 3 only)*  MILK *(Experiments 1a, 2a, 2a, and 2b)*  JUICE *(Experiments 1a, 2a, and 2b)*  Does Emma like milk? |
| List Sentence  2-noun  3-noun  4-noun  5-noun  Reference Sentence  Anaphor  No Anaphor  Probe Word  Referent  Distractor  Comprehension | Benjamin collected chairs, and his apartment had …  a bench and a barstool.  a recliner, a bench, and a barstool. *(Experiments 1a and 1b only)*  a rocker, a recliner, a bench, and a barstool. *(Experiments 1a and 1b only)*  an armchair, a rocker, a recliner, a bench, and a barstool.  He usually sat on the tall seat while he ate.  His home was very unique compared to others. *(Experiment 3 only)*  BARSTOOL *(Experiments 1a, 2a, 2a, and 2b)*  BENCH *(Experiments 1a, 2a, and 2b)*  Did Benjamin favor the barstool? |
| List Sentence  2-noun  3-noun  4-noun  5-noun  Reference Sentence  Anaphor  No Anaphor  Probe Word  Referent  Distractor  Comprehension | Gavin’s favorite ice cream flavors are …  chocolate and strawberry.  peppermint, chocolate and strawberry. *(Experiments 1a and 1b only)*  toffee, peppermint, chocolate and strawberry. *(Experiments 1a and 1b only)*  vanilla, toffee, peppermint, chocolate and strawberry.  He generally buys the pink ice cream during the summer.  He was often teased for his strong opinioned sweet tooth. *(Experiment 3 only)*  STRAWBERRY *(Experiments 1a, 2a, 2a, and 2b)*  CHOCOLATE *(Experiments 1a, 2a, and 2b)*  Did Gavin dislike strawberry ice cream? |
| List Sentence  2-noun  3-noun  4-noun  5-noun  Reference Sentence  Anaphor  No Anaphor  Probe Word  Referent  Distractor  Comprehension | Charlotte likes to make her own clothes using …  lace and spandex.  cotton, lace, and spandex. *(Experiments 1a and 1b only)*  polyester, cotton, lace, and spandex. *(Experiments 1a and 1b only)*  wool, polyester, cotton, lace, and spandex.  She doesn’t like to use too much of the stretchy material around her waist.  She would often look at magazines to be inspired by the new hottest trends. *(Experiment 3 only)*  SPANDEX *(Experiments 1a, 2a, 2a, and 2b)*  LACE *(Experiments 1a, 2a, and 2b)*  Is Charlotte sparing with spandex? |
| List Sentence  2-noun  3-noun  4-noun  5-noun  Reference Sentence  Anaphor  No Anaphor  Probe Word  Referent  Distractor  Comprehension | After graduation, Connor drank …  absinthe and tequila.  whiskey, absinthe, and tequila. *(Experiments 1a and 1b only)*  rum, whiskey, absinthe, and tequila. *(Experiments 1a and 1b only)*  vodka, rum, whiskey, absinthe, and tequila.  He overdid it with the Mexican alcohol during the celebration.  He had fun celebrating the start of a new chapter in his life. *(Experiment 3 only)*  TEQUILA *(Experiments 1a, 2a, 2a, and 2b)*  ABSINTHE *(Experiments 1a, 2a, and 2b)*  Did Connor drink too much absinthe? |
| List Sentence  2-noun  3-noun  4-noun  5-noun  Reference Sentence  Anaphor  No Anaphor  Probe Word  Referent  Distractor  Comprehension | While out bird watching, Ella spotted …  a vulture and a cardinal.  a dove, a vulture, and a cardinal. *(Experiments 1a and 1b only)*  a pigeon, a dove, a vulture, and a cardinal. *(Experiments 1a and 1b only)*  an eagle, a pigeon, a dove, a vulture, and a cardinal.  She was most impressed by the red bird on the tree.  She could always be found spending time with nature. *(Experiment 3 only)*  CARDINAL *(Experiments 1a, 2a, 2a, and 2b)*  VULTURE *(Experiments 1a, 2a, and 2b)*  Did Ella like robins? |
| List Sentence  2-noun  3-noun  4-noun  5-noun  Reference Sentence  Anaphor  No Anaphor  Probe Word  Referent  Distractor  Comprehension | For dinner this week, Addison bought …  tuna and lasagna.  soup, tuna, and lasagna. *(Experiments 1a and 1b only)*  casserole, soup, tuna, and lasagna. *(Experiments 1a and 1b only)*  meatloaf, casserole, soup, tuna, and lasagna.  On Wednesday she prepared the Italian dinner in her oven.  It was always pretty late by the time she got home to eat. *(Experiment 3 only)*  LASAGNA *(Experiments 1a, 2a, 2a, and 2b)*  TUNA *(Experiments 1a, 2a, and 2b)*  Did Addison cook tuna? |
| List Sentence  2-noun  3-noun  4-noun  5-noun  Reference Sentence  Anaphor  No Anaphor  Probe Word  Referent  Distractor  Comprehension | In art class, Elijah practiced working with …  pencil and paint.  charcoal, pencil, and paint. *(Experiments 1a and 1b only)*  marker, charcoal, pencil, and paint. *(Experiments 1a and 1b only)*  pen, marker, charcoal, pencil, and paint.  His favorite was the wet utensil brushed on canvas.  He loved creating masterpieces to show to his mom. *(Experiment 3 only)*  PAINT *(Experiments 1a, 2a, 2a, and 2b)*  PENCIL *(Experiments 1a, 2a, and 2b)*  Did Elijah prefer painting? |
| List Sentence  2-noun  3-noun  4-noun  5-noun  Reference Sentence  Anaphor  No Anaphor  Probe Word  Referent  Distractor  Comprehension | Oliver's empty fridge contained only …  mustard and ketchup.  mayonnaise, mustard, and ketchup. *(Experiments 1a and 1b only)*  relish, mayonnaise, mustard, and ketchup. *(Experiments 1a and 1b only)*  horseradish, relish, mayonnaise, mustard, and ketchup.  Plus he was almost out of the red condiment in the bottle.  He always dreaded having to deal with the grocery store. *(Experiment 3 only)*  KETCHUP *(Experiments 1a, 2a, 2a, and 2b)*  MUSTARD *(Experiments 1a, 2a, and 2b)*  Did Oliver have a lot of ketchup? |
| List Sentence  2-noun  3-noun  4-noun  5-noun  Reference Sentence  Anaphor  No Anaphor  Probe Word  Referent  Distractor  Comprehension | At the dog show, Elizabeth entered …  a mastiff and a Chihuahua.  a Rottweiler, a mastiff, and a Chihuahua. *(Experiments 1a and 1b only)*  a collie, a Rottweiler, a mastiff, and a Chihuahua. *(Experiments 1a and 1b only)*  a poodle, a collie, a Rottweiler, a mastiff, and a Chihuahua.  She won first place with the smallest dog in the competition.  She loved travelling the country to different shows every year. *(Experiment 3 only)*  CHIHUAHUA *(Experiments 1a, 2a, 2a, and 2b)*  MASTIFF *(Experiments 1a, 2a, and 2b)*  Did Elizabeth win with the Chihuahua? |
| List Sentence  2-noun  3-noun  4-noun  5-noun  Reference Sentence  Anaphor  No Anaphor  Probe Word  Referent  Distractor  Comprehension | Throughout his life, Alexander had worked as …  a carpenter and a fireman.  a driver, a carpenter, and a fireman. *(Experiments 1a and 1b only)*  a barber, a driver, a carpenter, and a fireman. *(Experiments 1a and 1b only)*  a waiter, a barber, a driver, a carpenter, and a fireman.  He did not like the dangerous career in his youth.  He was well known for his many different skills. *(Experiment 3 only)*  FIREMAN *(Experiments 1a, 2a, 2a, and 2b)*  CARPENTER *(Experiments 1a, 2a, and 2b)*  Did Alexander like firefighting? |
| List Sentence  2-noun  3-noun  4-noun  5-noun  Reference Sentence  Anaphor  No Anaphor  Probe Word  Referent  Distractor  Comprehension | Aiden, a hat aficionado, owned …  a tophat and a beret.  a visor, a top hat, and a beret. *(Experiments 1a and 1b only)*  a beanie, a visor, a tophat, and a beret. *(Experiments 1a and 1b only)*  a ball cap, a beanie, a visor, a tophat, and a beret.  He usually wore the French hat on his head.  He was always worried about his appearance. *(Experiment 3 only)*  BERET *(Experiments 1a, 2a, 2a, and 2b)*  TOPHAT *(Experiments 1a, 2a, and 2b)*  Did Aiden own a beret? |
| List Sentence  2-noun  3-noun  4-noun  5-noun  Reference Sentence  Anaphor  No Anaphor  Probe Word  Referent  Distractor  Comprehension | For Arbor Day, Lucas planted …^[[1]](#footnote-1)^  a willow and a maple.  a birch, a willow, and a maple.  a spruce, a birch, a willow, and a maple.  an oak, a spruce, a birch, a willow, and a maple.  He had a hard time finding the Canadian tree in the forest.  *--*  MAPLE  WILLOW  Did Lucas plant a tree for a special occasion? |

Appendix B

Details of analysis and results from reading time on list sentences.

The same methods were used to transform and identify outliers in the list-sentence reading times as in the reference-sentence reading times. This resulted in 12.7% of the trials being excluded from further analysis in Experiment 1a, 12.2% in Experiment 1b, 14.5% in Experiment 2a, and 11.7% in Experiment 3.

In general, per-character reading time on the list sentence increased as the number of nouns in the sentence increased. In Experiment 1a, the linear trend was significant *F*_1_(1, 84) = 12.14, *p* = .001, *F*_2_(1, 30) = 10.87, *p* = .003, $\eta_{p}^{2}$= .13, with no significant higher-order trends. In Experiment 1b, the linear trend was also significant, *F*_1_(1, 54) = 11.46, *p* = .001, *F*_2_(1, 30) = 6.99, *p* = .01, $\eta_{p}^{2}$= .18, with no significant higher-order trend.

In Experiment 2a, a paired-samples *t*-test revealed that per-character reading times were significantly faster in the two noun condition (*M* = 76.7, *SE* = 2.4) than in the five noun condition (*M* = 82.0, *SE* = 2.5), *t*_1_(68) = 3.23, p = .002, *t*_2_(29) = 3.91, *p* = .001, *d* = 0.26. Finally, in Experiment 3, a paired-samples *t*-test revealed that per-character reading times were significantly faster in the two-noun condition (*M* = 70.7, *SE* = 2.0) than in the five-noun condition (*M* = 73.7, *SE* = 2.4) in the subjects analysis, *t*_1_(64) = 2.28, *p* = .026, *d* = 0.17, but this effect was non-significant in the items analysis, *t*_2_(29) = 1.33, *p* = .19.

Appendix C

Counterbalancing order effects results and discussion.

The noun-order factor was included in all analyses in Experiments 1a and 1b. The only effects of order were the following: There was a significant main effect of order in the probe RT analysis in the items analysis in Experiment 1a, *F*_2_(1, 28) = 7.67, *p* =.01, but it did not interact with number of nouns. There was an order by number of nouns interaction in the accuracy analysis of Experiment 1b, *F*_2_(3, 90) = 4.46, *p* = .01; this interaction was due to the effect of the number of nouns being stronger for order 1 than for order 2, although the linear trend of number of nouns was significant for both orders (*p* < .001 with $\eta_{p}^{2}$ = .58 for order 1, and *p* = .006 with $\eta_{p}^{2}$ = .22 for order 2). Finally, there was a significant effect of order in Experiment 1b's probe RT analysis, *F*_2_(1, 30) = 10.72, *p* =.003, but it did not interact with number of nouns.

1. This item was only used in Experiments 1a and 1b. [↑](#footnote-ref-1)
